# Streamlined Multimodal DESI and MALDI Mass Spectrometry Imaging on a Singular Dual-Source FT-ICR Mass Spectrometer

**DOI:** 10.3390/metabo11040253

**Published:** 2021-04-20

**Authors:** Kevin J. Zemaitis, Alexandra M. Izydorczak, Alexis C. Thompson, Troy D. Wood

**Affiliations:** 1 Department of Chemistry, Natural Sciences Complex, University at Buffalo, State University of New York, Buffalo, NY 14260, USA; kevinzem@buffalo.edu (K.J.Z.); aizydorc@buffalo.edu (A.M.I.); 2 Department of Psychology, Park Hall, University at Buffalo, State University of New York, Buffalo, NY 14260, USA; act2@buffalo.edu

**Keywords:** multimodal imaging, Fourier transform ion cyclotron resonance (FT-ICR), mass spectrometry imaging (MSI), 3D printed/printing, desorption electrospray ionization (DESI), matrix-assisted laser desorption/ionization (MALDI), lipidomics

## Abstract

The study of biological specimens by mass spectrometry imaging (MSI) has had a profound influence in the various forms of spatial-omics over the past two decades including applications for the identification of clinical biomarker analysis; the metabolic fingerprinting of disease states; treatment with therapeutics; and the profiling of lipids, peptides and proteins. No singular approach is able to globally map all biomolecular classes simultaneously. This led to the development of many complementary multimodal imaging approaches to solve analytical problems: fusing multiple ionization techniques, imaging microscopy or spectroscopy, or local extractions into robust multimodal imaging methods. However, each fusion typically requires the melding of analytical information from multiple commercial platforms, and the tandem utilization of multiple commercial or third-party software platforms—even in some cases requiring computer coding. Herein, we report the use of matrix-assisted laser desorption/ionization (MALDI) in tandem with desorption electrospray ionization (DESI) imaging in the positive ion mode on a singular commercial orthogonal dual-source Fourier transform ion cyclotron resonance (FT-ICR) instrument for the complementary detection of multiple analyte classes by MSI from tissue. The DESI source was 3D printed and the commercial Bruker Daltonics software suite was used to generate mass spectrometry images in tandem with the commercial MALDI source. This approach allows for the generation of multiple modes of mass spectrometry images without the need for third-party software and a customizable platform for ambient ionization imaging. Highlighted is the streamlined workflow needed to obtain phospholipid profiles, as well as increased depth of coverage of both annotated phospholipid, cardiolipin, and ganglioside species from rat brain with both high spatial and mass resolution.

## 1. Introduction

Mass spectrometry imaging (MSI) has seen widespread use in the world of clinical mass spectrometry (MS) in part due to the flexibility of the label-free nature of the analysis, with applications ranging from biomarker discovery, studying metabolic pathways for pharmaceutical drugs, and spatial profiling of biomolecules [1,2,3,4]. With recent breakthroughs in prognostic and diagnostic applications, these analyses still require orthogonal confirmation despite high-resolution/accurate mass (HR/AM) analysis [5]. This has created many combined imaging approaches—known in the field as multimodal or multimode imaging, providing a secondary or tertiary round of analysis of the same (or serial) section [6]. Many have identified the tandem utility of comprehensive mapping of biomolecules from MSI, with targeted assays having incorporated multiple ionization sources or MSI methods [7,8,9,10], combination of MSI with microscopy and spectroscopy [11,12,13,14,15,16], and subsequent microdissection and extraction for liquid chromatography [17,18]. In light of these developments, two techniques of MSI have become predominant in the field, matrix-assisted laser desorption/ionization (MALDI) and desorption electrospray ionization (DESI), both of which are morphologically compatible [7,19].

While MALDI is still the most commonly implemented commercial form of MSI, the technique suffers from low ionization efficiencies of small molecules, such as neurotransmitters, and complex sample preparation steps for larger peptides and proteins [20,21,22]. In addition, MALDI has a prerequisite step prior to analysis requiring the deposition of a thin, homogenous layer of small organic acids (among other molecules) known as the matrix. The matrix is required for enhancement of ionization, and efficient absorption and distribution of laser energy; however, this can also create many isobaric clusters interfering with low mass-to-charge (*m*/*z*) analytes of interest [23]. Ambient ionization techniques, on the other hand, were introduced after laser desorption and require no such sample preparation; moreover, the matrix suppression effects observed within MALDI are not present with ambient ionization methods. However, analysis of large biomolecules from tissue by DESI has not been readily adopted without various forms of ion mobility spectroscopy (IMS) or modifications to commercial platforms [24,25], leading to the formation of a strong comprehensive multimodal approach for biomolecular imaging when DESI and MALDI are used in tandem. This has been shown by DESI-MSI of lipids and subsequent MALDI-MSI on the same tissue section for proteins, as well as increased coverage of lipids and gangliosides when utilized in tandem [26,27].

With a major application of both DESI and MALDI imaging in spatial lipidomics, with increasing interest in imaging of large biomolecules, HR/AM platforms with sufficient mass resolving power (RP) have become essential for proper clinical annotation of MS peaks [28,29,30]. Early MALDI analyses often suffered from lower mass RP and mass accuracy from the broad initial energy distributions and mass-independent velocities within time of flight (TOF) MS [31]. Although minimized within Fourier transform MS (FT-MS) platforms, this still does have an effect on analyses. Concerning lipidomic imaging analyses, sufficient RP is required for proper annotation and differentiation of phospholipids such as phosphatidylethanolamines (PE), phosphatidylcholines (PC), and phosphatidylserines (PS) as monoisotopic and isotopic peaks (A+1, A+2, etc.) commonly result in near isobars from varying levels of saturation on acyl chains (^12^C_1_ versus ^1^H_12_). More common is the varying cationic adduction forming near isobars, and coalescence of the monoisotopic and the A+2 isotopic peak of a lipid containing one less double bond (^12^C_2_^1^H_2_ versus ^13^C_2_) [32]. For example, two separate adducts of sodiated PS 36:1 and protonated PC 38:4 (Δ2.4 mDa) were only resolved after advanced absorption-mode Fourier transform (aFT) processing during a magnitude-mode MALDI Fourier transform ion cyclotron resonance (FT-ICR) imaging experiment [33]. Another example comes from the resolution of monoisotopic peaks of sodiated PC 34:1 from the protonated PC 36:4 (Δ2.5 mDa), which was resolved with advanced external data acquisition and aFT by DESI FT-ICR MSI [28]. The requisite mass RP in excess of 180 K at *m*/*z* 800 is capable only by FT-ICR mass analyzers, and certain Orbitraps [32], where multimodal DESI and MALDI imaging of murine brain on the same instrument has been reported on a TOF-MS capable of RP of 60 K at *m*/*z* 956 [26]. Therefore, the implementation of DESI (or other ambient ionization sources) and MALDI imaging on a singular orthogonal dual-source FT-ICR MS will be highly beneficial, as one does not have to exchange the ionization source or utilize multiple commercial or third-party software packages for image generation.

The sparse reporting of ambient MSI by FT-ICR is largely due to the low duty cycle and scanning rate of the mass analyzer, which is requisite to the unparalleled mass RP and has historically paired well with pulsed ionization sources [34]. To our knowledge, there are no reports of multimodal DESI and MALDI MSI on a single FT-MS platform, although multimodal MALDI and infrared matrix-assisted laser desorption electrospray ionization (IR-MALDESI) MSI has been reported on two separate FT-ICR MS instruments [35]. A need also exists for open-source software packages which can either handle several terabytes of unprocessed data or process reduced data files. To overcome the principal issue of requisite long detection periods, current lower-field instruments utilize external aFT post-processing techniques over traditional magnitude-mode Fourier transform (mFT) processing [33], with newer generations of instruments having dynamically harmonized and frequency multiplied ICR cells which allow for up to four-fold increases in acquisition speeds and gains in signal-to-noise ratio (SNR) and mass RP [36]. Despite these advances, few commercial or open-source software packages have been demonstrated for FT-MS imaging to resolve this secondary issue of image generation from ambient FT-ICR MS analyses without further development or substantial investment. We herein demonstrate the reprocessing of ambient serial acquisitions into a serial MALDI-MSI analysis using the native software to the MS platform for MALDI imaging. This streamlined approach allows for completion of multimodal imaging on a commercial Bruker Daltonics SolariX FT-ICR MS utilizing only the commercial suite of software for MALDI-MSI and the included advanced acquisition programs, allowing for a comprehensive global mapping of lipids and chemical species without mass analyzer bias and with an observed mFT RP routinely above 100 K at *m*/*z* 798 at a sampling rate of approximately 1 Hz.

## 2. Results

### 2.1. DESI FT-ICR MSI Image Generation

**Figure 1:**
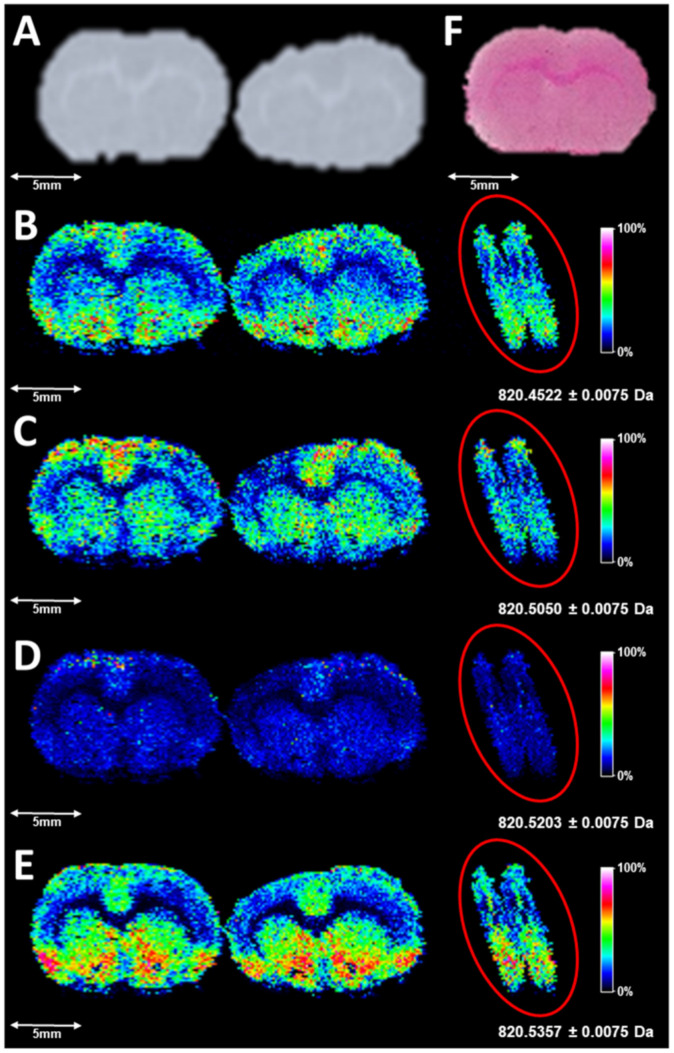
(**A**) is a digitized scan of the sections profiled by DESI-MSI, with corresponding images for the following *m*/*z* windows: (**B**) (820.4522 ± 0.0075), (**C**) (820.5050 ± 0.0075), (**D**) (820.5203 ± 0.0075), and (**E**) (820.5357 ± 0.0075). Images are created from inputting the transient of a DESI serial acquisition into the MALDI-MSI data file, reprocessing in FTMS Processing 2.2.0 (Bruker Daltonics, Bremen, Germany), and proper alignment of pixels in the sequence in FlexImaging 5.0 (Bruker Daltonics, Bremen, Germany). Highlighted to the right of the images in (**B**–**E**) are the artifacts produced from continuously scanning during the fly back and step. Line scans are aligned post-manual triggering of acquisition in FTMS Control 2.1.150 and subsequently sending the file of ASC II commands to the positioners. (**F**) represents an H&E-stained serial section. In the images, the highest ion intensities are white and the lowest ion intensities are black.

Utilizing a fly back pattern for DESI FT-ICR MSI (as shown in Supplementary Figure S1) 150 µm square pixels were created after determination of the average scan rate. A meandering pattern, which has been demonstrated to acquire line scans in the positive and negative direction, has been utilized in many other lab-built DESI-MSI sources [37]. This imaging pattern could not be utilized in this workflow due to the requirement of coordinating DESI scans with pixel coordinates in MALDI-MSI FlexImaging sequences of serial sections, which employs a fly back pattern, and is ultimately utilized for DESI-MSI image generation. The transient swapping procedure for DESI-MSI image generation is explained in greater detail for readers in Supplementary Information. Briefly, each resultant line of pixels corresponds to both the analytical line scan (left-to-right sampling), fly back (right-to-left movement), and a line step. The DESI images in Figure 1 are the resolved ions at different *m*/*z* values near the potassiated adduct of PC 36:4 (*m*/*z* 850.5203). Figure 1B–E also all show pixels that start from the measurement region on the left, ending after the artifact which is produced from fly back motion due to the continuous scanning before the subsequent start of the next line scan. This artifact can also be analytical in the reverse direction, as long as time per line scan is accounted for in the MALDI measurement region.

This continuous acquisition requires the proper alignment of the starting pixel of each line scan, as there will always be a delay either from manual triggering or contact closure, which varies for each analysis. Hence, the movement command 1MV-4 is the chosen alignment tool in the ASCII command file sequence sent from the Agilis AG-UC2 applet (Newport Corporation, Newport, CA, USA), shown in the fly back pattern in Supplementary Figure S1. This command triggers the sampling stage to move at a set step amplitude to the electronic limit switch, initiating the line scan. The proper number of pixels must be translated into a MALDI measurement region for high-fidelity DESI-MSI, and are created from MALDI-MSI sequences in this workflow; certain corrections are needed post-processing to account for variable timing of each FT-ICR scan which causes an alteration in the number of pixels per line scan. This average scan rate was studied post-analysis and calculated at an average of 1.128 s/scan, with variation over the 15,347 scans in the DESI serial acquisition acquired under 99% data reduction with the current acquisition server and accumulation during detection (ADD) pulse program in FTMS Control 2.1.150 (Bruker Daltonics, Bremen, Germany). This is a consideration for DESI analysis and does have a consequence, though it should be stated that the variable scanning rate does not matter for MALDI FT-ICR acquisition with pulsed ionization and detection. The encoded coordinates correspond directly to singular scans within the serial acquisition and can have tens of milliseconds of overhead time for analog to digital conversion and real-time display. However, the ion images produced are still representative of the imaged tissue section when a pulsed source is swapped for a continuous source.

This is possible due to the presence of an advanced pulse program within the commercial Bruker Daltonics software which allows for parallel external collection of ions in a multipole, while injected packets of ions are being analyzed in the ICR cell. External trapping of ions prior to simultaneous injection into the ICR cell has been demonstrated previously by DESI-MSI on a ThermoFisher LTQ FT Classic to dramatically increase the throughput of analysis, as well as doubling the scanning rate and boosting the SNR and duty cycle [28]. Furthermore, ADD has had previous use on a 12T Bruker Daltonics SolariX FT-ICR MS for negative electron transfer dissociation experiments with IMS to dramatically reduce experimental time [38]. Indeed, this increase in duty cycle allows for more comparable imaging times to DESI-MSI on Orbitrap FT-MS platforms, which routinely perform hybrid aFT and mFT transformation to the transient with parallel ion accumulation [39].

### 2.2. Comparison of DESI FT-ICR MSI to MALDI FT-ICR MSI

**Figure 2:**
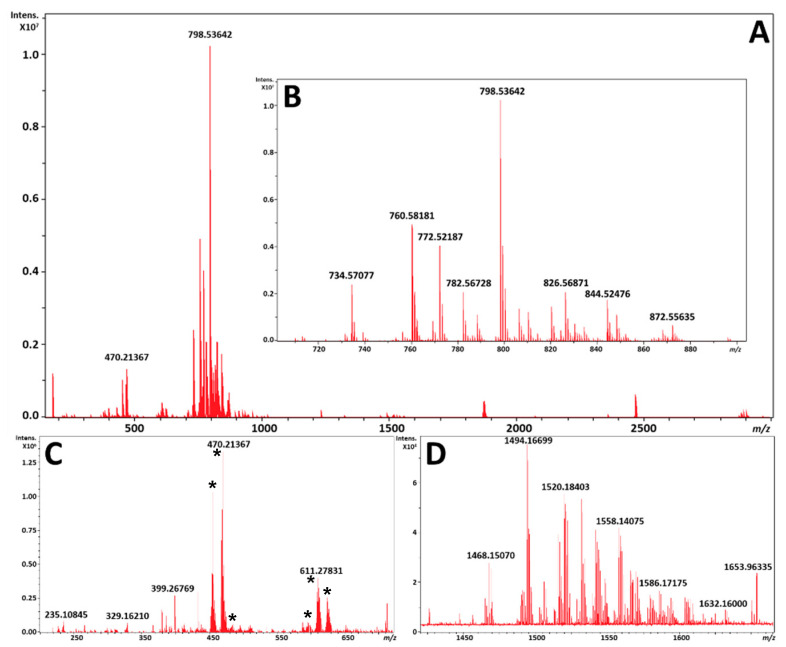
(**A**) is an exported average spectrum of every pixel from MALDI-MSI from FlexImaging 5.0 (Bruker Daltonics, Bremen, Germany). (**B**) is a zoomed view of the phospholipid profile from *m*/*z* 700 to 900. (**C**) is a zoomed view highlighting the matrix clusters formed from 1,5-DAN in the positive ion mode from *m*/*z* 200 to 725 marked by asterisks. (**D**) is a zoomed view of the putative cardiolipin and ganglioside profile from *m*/*z* 1425 to 1675.

**Figure 3:**
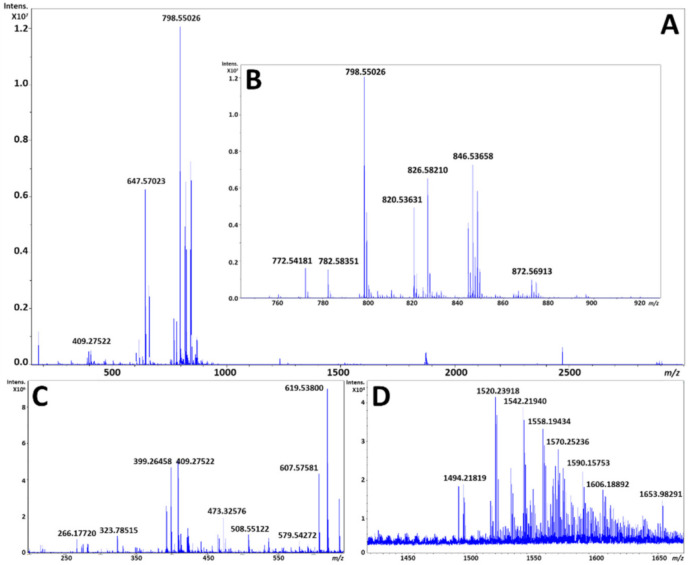
(**A**) is an exported average spectrum of every pixel from DESI-MSI from FlexImaging 5.0 (Bruker Daltonics, Bremen, Germany), (**B**) is a zoomed view of the phospholipid profile from *m*/*z* 700 to 900, (**C**) is a zoomed view highlighting the matrix cluster formed from 1,5-DAN in the positive ion mode from *m*/*z* 200 to 725, and (**D**) is a zoomed view of the putative cardiolipin and ganglioside profile from *m*/*z* 1425 to 1675.

The averaged mass spectrum from all pixels for two serial sections by MALDI and DESI each, respectively, is shown in Figure 2 and Figure 3. As can be expected, the MALDI process produces matrix clusters of 1,5-DAN in the positive ion mode (Figure 2C), where DESI does not exhibit these ions (Figure 3C) and has many distinct species which have localizations in the brain within that region. It is worth noting that FT-ICR MS does exhibit a TOF bias during transport of ions from source to detector, so an analysis targeting the lower *m*/*z* species would have to be performed separately for best results. However, the focus of these analyses was the phospholipid profiles, which despite being unique to each modality were highly complementary, with a base peak corresponding to a potassiated adduct of PC 32:0 in both ionization modes; it is interesting to note that each fingerprint was unique in the primary adduct formed for each PC, PE, and sphingomyelin (SM) noted. This is likely due to the tunneling nature of the laser beam through the sample to the glass substrate by MALDI, versus a surface ionization that is heavily impacted by extracellular concentrations of sodium and potassium at the surface.

**Figure 4:**
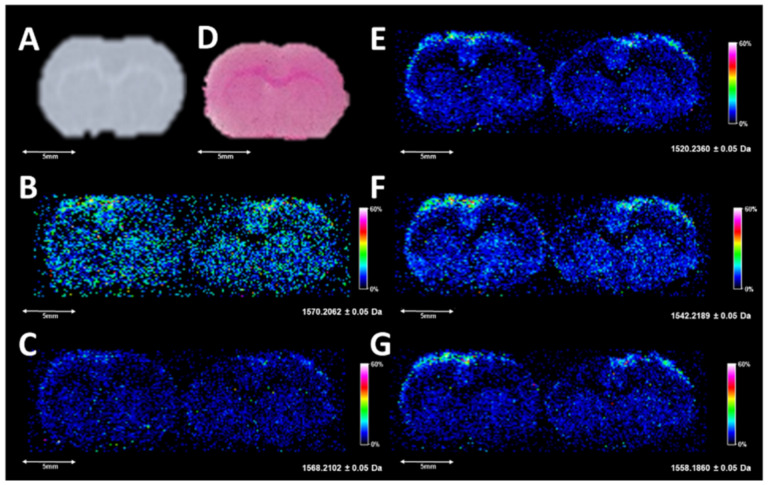
(**A**) is a digitized scan of the left section profiled by DESI-MSI, (**D**) is a completed H&E-stained serial section collected 220 µm from the DESI sampled tissue, with (**B**) (1570.2060 ± 0.05), (**C**) (1568.2100 ± 0.05), (**E**) (1520.2360 ± 0.05), (**F**) (1542.2190 ± 0.05), and (**G**) (1558.1860 ± 0.05) corresponding to the top five images in a *m*/*z* window from 1400 to 1600. In the images, the highest ion intensities are white and the lowest ion intensities are black.

Several species were also observed with similar abundance within the *m*/*z* range of 1400 to 1600 (Figure 2D and Figure 3D) which are annotated as cardiolipins (CL) and gangliosides; both have been previously observed and studied by MALDI and DESI [26,40,41]. Annotations of these species based upon HR/AM were completed in LIPID MAPS and METLIN, and putative matches with mass error calculations based upon adducts from LIPID MAPS are highlighted in Supplementary Tables S1 and S2. The DESI images of several of these species are shown in Figure 4.

**Figure 5:**
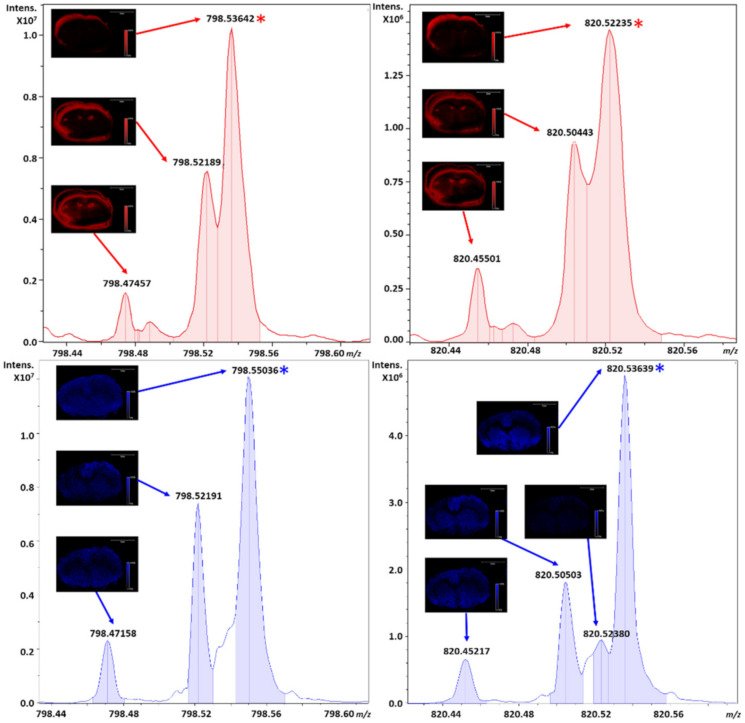
Individual zoomed views from the averaged spectra of all pixels of the potassiated adducts of PC 32:0 and PC 36:4 from *m*/*z* ranges of 794.42 to 798.61 and 820.43 to 820.57, respectively. This area of 210 mDa contains several species annotated with varying resolution from MALDI (top, red) and DESI (bottom, blue). The PC main adduct is marked with an asterisk for each respective peak.

When looking at several nearly isobaric lipid species by MALDI and DESI, distinct differences can be noted primarily for peak intensities of the various adducts of phospholipids as well as the peak resolution and width from the averaged spectra. Although RP depends on a myriad of factors including distribution of ions, repulsion in the cell, nearest neighboring peak intensities, and the duration of detection, analysis with the same ion transfer, excitation, and detection parameters within the Infinity cell of the FT-ICR enables a differential analysis of the sources which are at atmospheric and intermediate pressure on the same instrument without mass analyzer bias of another instrumental platform. Shown in Figure 5 is the zoomed view of all averaged pixels, highlighting the monoisotopic peaks correlating to the potassiated adducts of PC 32:0 and PC 36:4 marked by asterisks for both MALDI and DESI.

For PC 32:0, the resolution in DESI from the averaged spectrum is experimentally measured at a RP of ~105 K, with nearly a twenty percent increase in peak intensity versus a RP of ~64 K by MALDI. An approximately twenty-five percent increase in peak intensity was also observed for the nearest neighboring peak at *m*/*z* 798.52, where a left shoulder can be noted on PC 32:0 during DESI experiments at approximately half the relative intensity to the valley between the isobars in MALDI. Within the same Figure 5, for PC 34:6, the RP of DESI was experimentally measured at ~103 K with an approximately five-fold increase in peak intensity over MALDI, versus a RP of only ~43 K by MALDI. Moreover, the nearest neighboring peak at *m*/*z* 820.50 increased in intensity approximately two-fold for DESI over MALDI.

This decrease in RP from the averaged pixels is not only resultant from an increase in ionization efficiency via DESI for the major species, but also coalescence of PC 32:0 and PC 36:4 with near isobars in the spectral averaging of all pixels. This coalescence not only impacts the experimentally observed RP, but shifts the centroid of the peak impacting mass accuracy. In order to obtain sufficient RP to resolve these peaks at fifty percent full width half maximum (FWHM), longer transient acquisitions and accumulations are required. However, for DESI, the differential ionization efficiencies and other factors for the adduct allowed for sufficient resolution to the nearest isobar during a moderate throughput analysis contrary to the other technique.

## 3. Discussion

Application of ambient MSI has been applied hundreds of times to faster scanning FT-Orbitrap platforms or to hybrid FT-ICR MS that accumulate ions externally within a multipole or ion traps [42,43,44,45]. In contrast, few reports exist of DESI imaging being applied to FT-ICR on commercial instrumentation; one report utilized a fully automated machined DESI source previously mentioned on a past generation of Bruker Daltonics instrumentation after recoding FlexImaging to control the DESI stage, instead of the MALDI on the commercial instrument allowing for a broad array of ambient sources to be applied [46]. A second report entailed the construction of a custom IR-MALDESI source on a ThermoFisher hybrid LTQ FT Ultra [47]. Additionally, the most recent report utilized a commercial DESI source with advanced external data acquisition, which made continuous line scanning DESI-MSI possible on a ThermoFisher hybrid LTQ FT Classic and highlighted the utility of external data acquisition, parallel accumulation and detection [28].

Certainly, the key to applying these ambient MSI techniques in a moderate to high-throughput manner is to use advanced data acquisition schemes, including ADD and advanced aFT processing, effectively fully saving either the entirety of the accumulation or detection period and recovering lost SNR and mass resolution from shorter transient acquisitions. In this approach, DESI imaging of an area of 27 mm by 10.4 mm took 287 min, and we demonstrated the swapping of the MALDI transient in one analysis for the DESI transient and reprocessing in FTMS Processing 2.2.0 (Bruker Daltonics, Bremen, Germany). Further streamlining the process from acquisition to data analysis utilizing the commercial software packages, which are already implemented in workflows for on-line recalibration of FT-ICR serial acquisitions, and aFT processing utilizing the built-in absorption-mode processing (AMP) tool. To note, MSI was not aFT processed within these comparisons; however, experimentally observed aFT mass RP exceeds 280 K at *m*/*z* 798.5 for DESI when proper phase calibration is applied, whereas mFT DESI routinely achieved ~100 K at *m*/*z* 798.5 and mFT MALDI routinely achieved ~60 K at *m*/*z* 798.5 for a 0.8386 s transient. The differences in resolution may be due to a multitude of factors that impact the analysis, but are outside of the scope of the presented novel workflow and expected results from each method.

During these experiments, the lipid fingerprint was confirmed to be expanded by utilizing both DESI and MALDI on serial sections, and the complementary effect of these two ionization sources has been well documented within the literature; and regardless of the potential for ultra-high mass RP with increased transient times, the analysis is in agreement with a previous comparative study of DESI and MALDI utilizing the swapping of sources on the same TOF instrument [26]. Contrary to this previous report, the process of multimodal MALDI and atmospheric pressure ionization (API) imaging is streamlined as the FT-ICR instrument used within this research has an orthogonal MALDI source to the API interface, allowing for the DESI and MALDI sources to be installed on the instrument at the same time, fully mitigating instrumental downtime between runs.

Within this study, phospholipid profiles were noted as unique to both MALDI and DESI, as demonstrated in Figure 2 and Figure 3. In addition, there were several areas of the spectra that were densely populated in DESI including regions below *m*/*z* 500 and above *m*/*z* 1200 which have potential for further optimization and higher sensitivity on FT-ICR MS. CLs and gangliosides were previously reported as difficult classes to analyze in common matrixes such as 1,5-DAN and others without prior sample preparation steps [41,48]. Moreover, MALDI analyses have a drop off for potential sensitivity once the sample has been fully destructively analyzed down to the glass substrate. These complementary fingerprints, highlighted in Figure 2D and Figure 3D, can be further enhanced through longer accumulation and detection periods as FT-ICR allows for several seconds of accumulation and detection of ions and DESI is non-destructive technique.

Utilizing 3D printing technologies in the production of the DESI source also allows for many other API and ambient imaging sources to be swapped onto the FT-ICR platform, with subtle modifications to the sampling stage and ionization source holder, allowing for further targeting of complementary classes of molecules through different modalities, as DESI and MALDI have been demonstrated to have similar utility. Ambient sources such as low-temperature plasma have been demonstrated in ambient MSI analysis, as well as having been fully 3D printed [49], and with many other applications in MS having been demonstrated by 3D printing, there is much potential for expansion [50]. Indeed, this is an area where rapid design and prototyping by computer modelling and subsequent 3D printing excels over conventional means of machining and manufacturing laboratory made sources and interfaces for MS.

Experiments of lipid imaging by DESI and subsequent peptide and protein profiling by MALDI have been demonstrated on the same section, utilizing DESI as a sample preparation step without loss of chemical information from lipid depletions [27]. The current setup of the 3D-printed DESI FT-ICR MS source utilizes instrumental power supplies to charge the capillary extension. It was noted that having a conductive ITO slide for DESI analysis *decreased* the signal potential by two orders of magnitude. This is due to the slide incurring the negative high voltage potential of the charged capillary extension in the positive ion mode, or vice versa in the negative ion mode, due to the unique application of the high voltage for electrospray in Bruker Daltonics MS platforms. Mitigation of this issue is possible through application of a positive potential applied to the emitter and spray solvent, although negating the negative charge sinking effect on ITO utilizing tens of milliamps of instrumental high voltage, the setup has been previously observed to decrease the efficiency of production and transmission of positive ions on this instrument [51]. Unless a non-conductive slide can be conductively coated subsequently after DESI analysis, MALDI of the same section will not be as effective for users trying to adapt Bruker Daltonics instruments to this methodology. Thus, gold sputtering and other methods applicable to electron microscopy may be needed for this purpose.

Utilizing this workflow, scanning during the return fly back movement to the right limit switch of the array also has potential for an additional analytical line scan that could be acquired in the *reverse direction* if set at the same velocity as the positive direction, allowing for the acquisition of a third and fourth DESI image of the same tissue in reverse, when using two brains per slide. Although tandem analysis of the same section by DESI and MALDI is not amenable due to the application of potential on the capillary, previous studies have shown that a wetting quill in front of the emitter increases the desorption of peptides and proteins from surfaces by allowing for extra time for efficient protein extraction from the thin film formed, and DESI itself has been utilized as a sample preparation method [27,52]. The emitter itself could be utilized in such a manner in the positive and negative direction, or even allow for transition of spray solvent composition or reactive chemistry post a controlled analysis without additional setup [53,54,55], making the approach of accounting for the fly back scans in the continual serial acquisition even more practical.

## 4. Materials and Methods

### 4.1. Chemicals

HPLC-grade methanol was from Fisher Scientific (Fair Lawn, NJ, USA), hydrofluoric acid was from JT Baker (Phillipsburg, NJ, USA), HPLC-grade water with 0.1% formic acid (*v*/*v*) was from Honeywell (Muskegon, MI, USA), xylene and 97% 1,5-diaminonapthalene (1,5-DAN, used without further purification) were from Aldrich Chemical (Milwaukee, WI, USA), and hematoxylin and eosin Y were from Sigma Aldrich (St. Louis, MO, USA).

### 4.2. Tissue Processing

All experiments were performed according to protocols approved by the University at Buffalo’s Institutional Animal Care and Use Committee (Protocol #PSY07031N). For these experiments, a rat brain was harvested from a Long–Evans (hooded) rat and rapidly frozen to −65 °C, allowed to warm to −20 °C, and then cryosectioned into 12 μm sections on a Thermo Fisher Scientific Microm International GmbH HM550 cryostat (Walldorf, Germany). Serial sections were taken and were thaw mounted onto indium tin oxide (ITO) slides from Bruker Daltonics (Billerica, MA, USA) for MALDI analysis and Corning plain microscope slides (Corning, NY, USA) for DESI analysis and stored at −65 °C until the experiment. All experiments were performed within several days post-sectioning. Hematoxylin and eosin staining (H&E) was completed in the following manner: 95% methanol, 70% methanol, 100% water for 30 s each, then hematoxylin for 3 min, then 100% water, 95% methanol, 100% methanol for 30 s each, then eosin for 1 min, then 95% methanol and 100% methanol for 30 s each, and 2 cycles of xylene for 1 min. The slide was desiccated and digitized using a digital scanner.

### 4.3. Instrumentation

An unmodified commercial Bruker Daltonics 12T SolariX FT-ICR MS equipped with an Infinity cell and orthogonal electrospray and MALDI source with a 1 kHz SmartBeam II frequency-tripled (355 nm) Nd: YAG laser (Bremen, Germany) was used for both the DESI and MALDI-MSI analysis. Both MALDI and DESI serial acquisitions were acquired with broadband excitation at a 2 megaword time domain from *m*/*z* 147.56 to 3000.00, with a free induction decay (FID) transient of 0.8389 s; the resultant mFT resolution is estimated at 190 K at *m*/*z* 400. For the purposes of these experiments, the ion optics and Infinity cell parameters were not altered in between DESI and MALDI for the purposes of direct comparison without bias.

### 4.4. DESI-FT-ICR MSI Analysis

**Figure 6:**
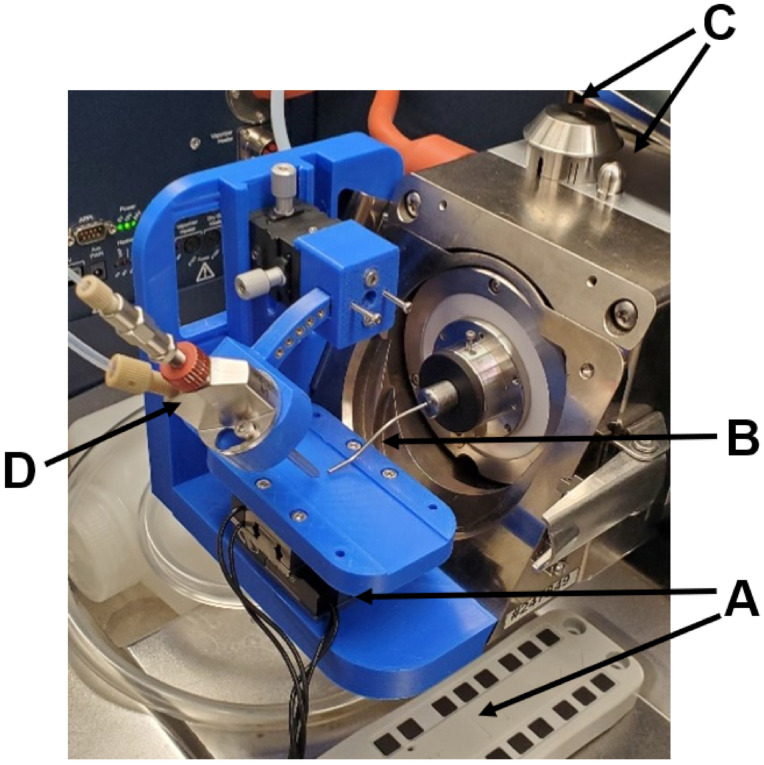
Photograph of the sourced mounted on the mass spectrometer, highlighting the Agilis LS-25-27 array and UC2 controller (**A**), the capillary extension and nanospray gas diverter (**B**), the removed endplate and capillary nub (**C**), and the standard sprayer electrospray emitter (**D**).

For DESI-MSI, a lab-built source through 3D printing, shown in Figure 6, was utilized and described in great depth elsewhere [51]. Briefly, an XY array of Agilis LS25-27 positioners and AG-UC2 controller (Newport Corporation, Newport, CA, USA) (Figure 6A) is externally controlled by a command file encoded in ASC-II and simultaneously triggered from the Agilis AG-UC2 controller applet (Newport Corporation, Newport, CA, USA) with acquisition of scans in FTMS Control 2.1.150 (Bruker Daltonics, Bremen, Germany); motion is tracked and the time per line scan is counted. The endplate and capillary nub (Figure 6C) are replaced with a capillary extension and Bruker nanospray gas diverter (Figure 6B), with the Bruker standard ESI sprayer (Figure 6D) being held by the 3D-printed body.

Sections were scanned at a linear velocity of 130 μm/s, with horizontal rows separated by a 150 μm step, in order to produce square 150 μm pixels. The ADD pulse program in FTMS Control 2.1.150 was used to optimize for an average scan of 1.128 s/scan, with 1.000 s accumulation of ions. A methanol–water solution was sprayed at a ratio of 9:1 with 0.1% formic acid (*v*/*v*) for DESI-MSI analysis at a solvent flow rate of 0.85 μL/min through a 0.5 µm porosity javelin filter (Thermo Scientific, Bellefonte, PA, USA) with 5.0 bar of nitrogen gas nebulizing pressure and −5.5 kV capillary voltage. The end plate voltage was set to 0 V during these experiments, and the end plate was removed as shown in Figure 6. The geometries were optimized with a 55° emitter incident angle 1.0 mm from the surface, and a 7° collection angle of the capillary extension less than 0.5 mm from the surface—and 5.0 mm from the electrospray emitter.

A Sutter Instrument P-2000 micropipette puller (Novato, CA, USA) was used to pull solvent capillaries from 200.0 μm outer diameter (OD) and 100.0 μm inner diameter (ID) fused silica (Polymicro Technologies, Phoenix, AZ, USA) to a measured 35 μm OD tip, as shown in Supplementary Figure S2, with the program for the P-2000 shown in Supplementary Table S3. The emitter was etched in a 10% hydrofluoric acid solution for 10 min following pulling of the emitter and checked under bright-field microscopy. The capillary was positioned within 1.0 mm of the capillary protruding the nebulizing gas channel of the standard sprayer.

### 4.5. MALDI-FT-ICR MSI Analysis

For MALDI-MSI, ITO slides were sublimed with a thin layer of 1,5-DAN via a lab-built sublimation chamber. Prior to sublimation, slides were placed in a desiccator to come to room temperature for 15 min while the sublimation chamber was heated to 140 °C. Once at room temperature, the slides were weighed, and two slides were simultaneously sublimed within the chamber for 20 min, with pressure stabilizing at 105 mTorr for 1,5-DAN. Post sublimation, the slides were desiccated prior to a second weighing for determination of an average coverage, and 150 μg/cm^2^ of 1,5-DAN was deposited. A digital scan was loaded into FlexImaging 5.0 (Bruker Daltonics, Bremen, Germany) to control the raster and acquisition of scans with spatial resolution set at 150 μm. The DESI method was utilized, and optimization of the instrument’s laser power was completed prior to imaging with 1000 shots per pixel at a 1 kHz repetition rate.

### 4.6. Image Generation, and Data Analysis for MSI

All MSI images were generated in FlexImaging 5.0 (Bruker Daltonics, Bremen, Germany) with a 0.95 ICR noise threshold (default), with data reduced to 96.5%. Image generation of DESI-MSI was carried out by swapping the transient of serial acquisition from DESI into the file folder of the MALDI-MSI serial acquisition. This data file was post-processed with FTMS Processing 2.2.0 (Bruker Daltonics, Bremen, Germany), and the file was saved into a copy of the MALDI FlexImaging sequence subdirectory which was acquired, accounting for the proper number and alignment of pixels for each line scan of the completed DESI acquisition. This process is explained in greater depth in Supplementary Information. After creation of images, average spectra were created by importing into DataAnalysis 5.0 (Bruker Daltonics, Bremen, Germany) for both DESI and MALDI analyses. Identification of species was carried out in LIPID MAPS (www.lipidmaps.org/ (accessed on 17 February 2021)) [56], and METLIN (www.metlin.scripps.edu/ (accessed on 17 February 2021)) [57].

## 5. Conclusions

Multimodal DESI and MALDI-MSI was demonstrated on a singular commercial dual-source FT-ICR instrument, without the need to exchange ionization sources or the application of external software to the existing commercial suite for MALDI-MSI. DESI FT-ICR MSI in this manner has the benefit of routine mFT RP above 100 K at *m*/*z* 798, and vast potential for RP several times higher than experimentally demonstrated. This is sufficient to resolve nearly all isobaric species at a 1 Hz scanning rate, which would coalesce into a singular peak in other lower-resolution MS platforms. Although the potential for spatial resolution of DESI is less than that of MALDI, especially when utilizing sublimation techniques for matrix application, complementary chemical information can be obtained from each ionization source at the same resolution in an orthogonal manner for confirmation of annotations. Unlike previous DESI FT-ICR MSI workflows, we used no modifications or third-party external software to the instrument, making this a streamlined approach for users to complete DESI, or other ambient modalities prior to MALDI-MSI. We also demonstrate the stability of utilizing 3D printing technologies for construction of the DESI source, which produce a sufficiently stable MSI platform that can be easily modified to adapt for other aforementioned sources for broader high-fidelity imaging purposes.

## Data Availability

The data presented in this study are available on request from the corresponding author, the datasets are not publically available due to size of the files.

## Supplementary Materials

The following are available online at https://www.mdpi.com/article/10.3390/metabo11040253/s1, Figure S1: DESI fly back pattern and line scan stage control, Figure S2: Bright-field image of the pulled and etched fused silica emitter, Table S1: annotations of DESI and MALDI cardiolipin profiles from LIPID MAPS, Table S2: annotations of DESI and MALDI phospholipids profiles from LIPID MAPS, Table S3: Micropipette puller program for the DESI fused silica emitter.

## Abbreviations

3D	three dimensional
MS	mass spectrometry/spectrometer
FT-MS	Fourier transform mass spectrometer/spectrometry
FT-ICR	Fourier transform ion cyclotron resonance
ADD	accumulation during detection
ICR	ion cyclotron resonance
AMP	absorption-mode processing
aFT	absorption-mode Fourier transform
mFT	magnitude-mode Fourier transform
SNR	signal-to-noise ratio
RP	resolving power
FWHM	full width half maximum
HR/AM	high-resolution/accurate mass
TOF	time of flight
IMS	ion mobility spectrometry
MSI	mass spectrometry imaging
DESI	desorption electrospray ionization
MALDI	matrix-assisted laser desorption/ionization
1,5-DAN	1,5-diaminonapthalene
ITO	indium tin oxide
H&E	hematoxylin and eosin
PC	phosphatidylcholine
PS	phosphatidylserine
PE	phosphatidylethanolamine
SM	sphingomyelin
CL	cardiolipin
