# Supplementary material for: Streamlined Multimodal DESI and MALDI Mass Spectrometry Imaging on a Singular Dual-Source FT-ICR Mass Spectrometer
Source: Metabolites. 2021 Apr 20;11(4):253. doi: 10.3390/metabo11040253 (PMC8073082; doi:10.3390/metabo11040253)
Supplement: Supplementary file 1 [file metabolites-11-00253-s001.zip › metabolites-1135650-supplementary.pdf]

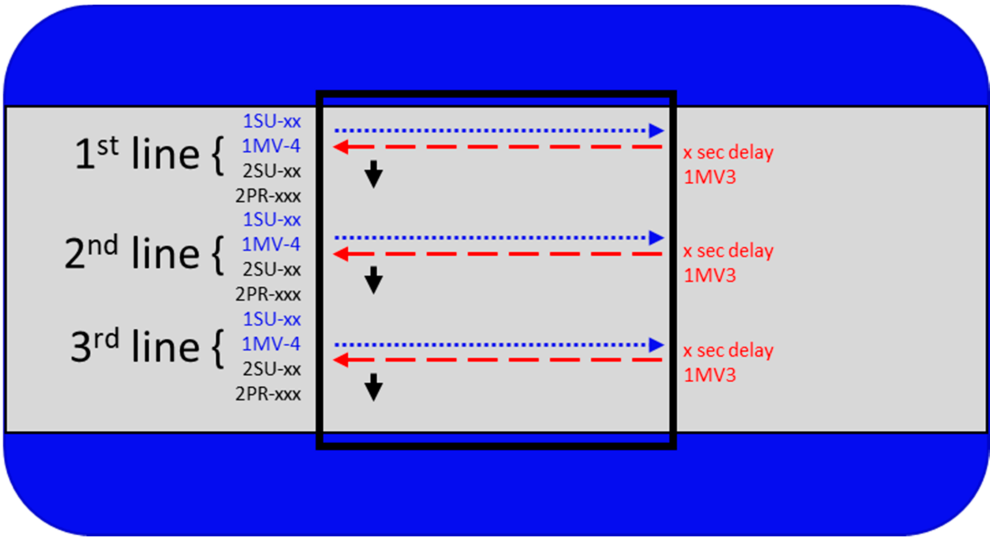

Supplementary Figure S1: Fly back pattern and subsequent ASCII commands utilized in the command file utilized for the XY array of Agilis LS-25-27 positioners. The blue rectangle with rounded corners is the 3D printed slide holder, gray rectangle is the slide (drawn to relative scale for 25mm by 75mm), with the black box representing the relative scale of the 27mm by 27mm travel range of the linear array. The ASCII commands functions are color coded blue, black, and red for the relative stage movement shown by the arrows and are as follows where 1 and 2 are the X or Y stages, and xx corresponds to an integer: 1SU-xx, set the negative step size of axis 1 to xx; 1MV-4, move to the negative limit (left limit switch) at the set step size; 1MV3, move to the positive limit switch at maximum step size and velocity after a set number of delays; 2SU-xx, set the negative step size of axis 2 to xx; 2PR-xxx, move axis two -xxx steps to the negative limit. Number of steps and step sizes for determination of average linear velocity must be calibrated prior to each run.

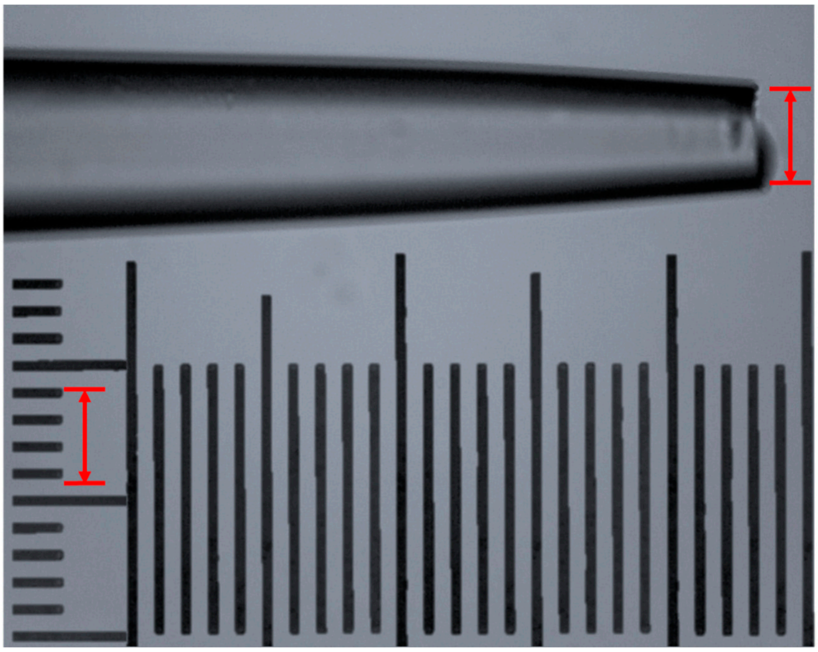

Supplementary Figure S2: Measurement of the pulled spray capillary by bright-field microscopy, each graduation in the scale bar is 10µm. The final outer diameter (OD) of the tip was estimated at 35µm after measurement of pixels, the jet of solvent produced allowed estimation of the inner diameter (ID) at 30um. Images were captured using an AmScope 1.3MP USB camera and AmScope 3.7 software.

Supplementary Table S1: Annotations of species from the averaged MALDI and DESI-MSI, non-recalibrated from LIPIDMAPS, peaks with greater than 0.075% intensity were taken from the charge deconvolution in DataAnalysis 5.0 and searched with parameters of ±0.1 Da as peaks were not internally recalibrated. The ionization source is indicated, observed mass, matched mass, delta change, annotation, name, and ionic formula are noted.

Ionization Source	Input Mass	Matched Mass	Delta (mDa)	Name	Formula
DESI	1520.2392	1520.1517	87.5	CL 76:1	C85H164O17P2
DESI	1542.2194	1542.1360	83.4	CL 78:4	C87H162O17P2
DESI	1542.2194	1542.1336	85.8	CL 76:1	C85H164O17P2Na
DESI	1558.1943	1558.1075	86.8	CL 76:1	C85H164O17P2K
DESI	1532.1727	1532.1880	-15.3	CL 78:0	C87H170O17P2
DESI	1532.1727	1532.0919	80.8	CL 74:0	C83H162O17P2K
DESI	1590.1575	1590.1312	26.3	CL 78:2	C87H166O17P2Na2
DESI	1494.2182	1494.1360	82.2	CL 74:0	C83H162O17P2
DESI	1516.1962	1516.1204	75.8	CL 76:3	C85H160O17P2
DESI	1516.1962	1516.1179	78.3	CL 74:0	C83H162O17P2Na
DESI	1592.2323	1592.1492	83.1	CL 80:3	C89H166O17P2Na
DESI	1592.2323	1592.1492	83.1	CL 80:4	C89H166O17P2Na
DESI	1592.2323	1592.1468	85.5	CL 78:1	C87H168O17P2Na2
DESI	1548.2695	1548.1830	86.5	CL 78:1	C87H168O17P2
DESI	1522.1372	1522.1673	-30.1	CL 76:0	C85H166O17P2
DESI	1522.1372	1522.1098	27.4	CL 78:5	C87H160O17P2
DESI	1589.6546	1589.5843	70.3	LPIM6 16:0	C61H109O42PNa2
DESI	1603.6668	1603.5999	66.9	LPIM6 17:0	C62H111O42PNa2
DESI	1529.6379	1529.7239	-86.0	PIM4 34:1	C67H121O33PNa2
DESI	1501.6075	1501.6950	-87.5	PIM4 34:4	C67H115O33PNa
DESI	1501.6075	1501.6926	-85.1	PIM4 32:1	C65H117O33PNa2
DESI	1500.1494	1500.1254	24.0	CL 76:2	C85H162O17P2
DESI	1551.6561	1551.6098	46.3	LPIM6 18:2	C63H109O42P
DESI	1551.6561	1551.7472	-91.1	PIM4 36:1	C69H125O33PK
DESI	1521.6566	1521.7003	-43.7	PIM4 34:2	C67H119O33PK
MALDI	1494.1670	1494.1360	31.0	CL 74:0	C83H162O17P2
MALDI	1494.1670	1494.0785	88.5	CL 76:5	C85H156O17P2
MALDI	1520.1840	1520.1517	32.3	CL 76:1	C85H164O17P2
MALDI	1520.1840	1520.0941	89.9	CL 78:6	C87H158O17P2
MALDI	1532.1250	1532.0578	67.2	CL 78:9	C87H152O17P2
MALDI	1532.1250	1532.1880	63.0	CL 78:0	C87H170O17P2
MALDI	1532.1250	1532.0553	69.6	CL 76:6	C85H154O17P2Na
MALDI	1532.1250	1532.0919	33.1	CL 74:0	C83H162O17P2K
MALDI	1532.1250	1532.0529	72.1	CL 74:3	C83H156O17P2Na2
MALDI	1558.1408	1558.1075	33.3	CL 76:1	C85H164O17P2K

MALDI	1558.1408	1558.0686	72.2	CL 76:4	C85H158O17P2Na2
MALDI	1542.1672	1542.1360	31.2	CL 78:4	C87H162O17P2
MALDI	1542.1672	1542.1336	33.6	CL 76:1	C85H164O17P2Na
MALDI	1566.1697	1566.1312	38.5	CL 76:0	C85H166O17P2Na2
MALDI	1568.1812	1568.1492	32.0	CL 78:2	C87H166O17P2Na
MALDI	1548.2160	1548.1830	33.0	CL 78:1	C87H168O17P2
MALDI	1506.1082	1506.0421	66.1	CL 76:8	C85H150O17P2
MALDI	1506.1082	1506.0397	68.5	CL 74:5	C83H152O17P2Na
MALDI	1506.1082	1506.0373	70.9	CL 72:2	C81H154O17P2Na2
MALDI	1586.1718	1586.1388	33.0	CL 78:1	C87H168O17P2K
MALDI	1586.1718	1586.0999	71.9	CL 78:4	C87H162O17P2Na2
MALDI	1516.1508	1516.1204	30.4	CL 76:3	C85H160O17P2
MALDI	1516.1508	1516.0628	88.0	CL 78:8	C87H154O17P2
MALDI	1516.1508	1516.1179	32.9	CL 74:0	C83H162O17P2Na
MALDI	1468.1507	1468.0628	87.9	CL 74:4	C83H154O17P2
MALDI	1490.1343	1490.1047	29.6	CL 74:2	C83H158O17P2
MALDI	1490.1343	1490.0472	87.1	CL 76:7	C85H152O17P2
MALDI	1554.1104	1554.0421	68.3	CL 80:12	C89H150O17P2
MALDI	1554.1104	1554.0397	70.7	CL 78:9	C87H152O17P2Na
MALDI	1554.1104	1554.0762	34.2	CL 76:3	C85H160O17P2K
MALDI	1554.1104	1554.0373	73.1	CL 76:6	C85H154O17P2Na2
MALDI	1621.8333	1621.7972	36.1	PIM5 32:0	C71H129O38P
MALDI	1584.8609	1584.9360	-75.1	Hex(3)-HexNAc-NeuAc-Cer 40:1;O2	C77H139N3O31
MALDI	1584.8609	1584.8608	0.1	Hex(3)-HexNAc-NeuGc-Cer 36:1;O2	C73H131N3O32Na
MALDI	1584.8609	1584.8398	21.1	Hex(3)-HexNAc-NeuAc-Cer 36:1;O2	C73H131N3O31K
MALDI	1584.8609	1584.9100	-49.1	Hex(3)-HexNAc(2)-Cer 42:2;O2	C76H137N3O28Na2
MALDI	1606.8485	1606.9333	.-84.8	Hex(3)-HexNAc(2)-Cer 44:2;O2	C78H141N3O28K
MALDI	1606.8485	1606.8427	5.8	Hex(3)-HexNAc-NeuGc-Cer 36:1;O2	C73H131N3O32Na2

Supplementary Table S2: Annotations of species from the averaged MALDI and DESI-MSI, non-recalibrated from LIPIDMAPS, peaks with greater than 0.075% intensity were taken from the charge deconvolution in DataAnalysis 5.0 and searched with parameters of ±0.005 Da as peaks were not internally recalibrated. The ionization source is indicated, observed mass, matched mass, delta change, annotation, name, and ionic formula are noted.

Ionization Source	Input Mass	Matched Mass	Delta (mDa)	Name	Formula
DESI	798.5503	798.5491	1.2	IPC 34:0;O3	C40H80NO12P
DESI	798.5503	798.5492	1.1	HexCer 36:1;O4	C42H81NO10K
DESI	846.5366	846.5372	-0.6	SHexCer 36:1;O3	C42H81NO12SNa
DESI	846.5366	846.5410	-4.4	PC 38:5	C46H82NO8PK
DESI	846.5366	846.5410	-4.4	PE 41:5	C46H82NO8PK
DESI	798.5219	798.5256	-3.7	PS 35:1	C41H78NO10PNa
DESI	798.5219	798.5198	2.1	PE dO-40:8	C45H78NO6PK
DESI	826.5821	826.5804	1.7	IPC 36:0;O3	C42H84NO12P
DESI	826.5821	826.5805	1.6	HexCer 38:1;O4	C44H85NO10K
DESI	848.5658	848.5623	3.5	IPC 36:0;O3	C42H84NO12PNa
DESI	846.5044	846.5046	-0.2	PE 40:6;O	C45H78NO9PK
DESI	798.4716	798.4682	3.4	PS 34:2	C40H74NO10PK
DESI	820.5050	820.5099	-4.9	PS 37:4	C43H76NO10PNa
DESI	772.5418	772.5463	-4.5	PS O-34:0	C40H80NO9PNa
DESI	826.5708	826.5721	-1.3	PE O-42:7	C47H82NO7PNa
DESI	826.5708	826.5723	-1.5	PC 36:1	C44H86NO8PK
DESI	826.5708	826.5723	-1.5	PE 39:1	C44H86NO8PK
DESI	820.5239	820.5253	-1.4	PE 39:4	C44H80NO8PK
DESI	820.5239	820.5253	-1.4	PC 36:4	C44H80NO8PK
DESI	874.5698	874.5685	1.3	SHexCer 38:1;O3	C44H85NO12SNa
DESI	874.5698	874.5723	-2.5	PC 40:5	C48H86NO8PK
DESI	874.5698	874.5652	4.6	Hex2Cer 32:0;O2	C44H85NO13K

DESI	844.5241	844.5253	-1.2	PC 38:6	C46H80NO8PK
DESI	844.5241	844.5253	-1.2	PE 41:6	C46H80NO8PK
DESI	826.4980	826.4995	-1.5	PS 36:2	C42H78NO10PK
DESI	820.4522	820.4525	-0.3	PS 36:5	C42H72NO10PK
DESI	874.5337	874.5357	-2.0	PC 42:11	C50H78NO8PNa
DESI	844.4480	844.4525	-4.5	PS 38:7	C44H72NO10PK
DESI	782.5482	782.5459	2.3	PE dO-40:8	C45H78NO6PNa
DESI	782.5482	782.5460	2.2	PC O-34:2	C42H82NO7PK
DESI	782.5482	782.5460	2.2	PE O-37:2	C42H82NO7PK
DESI	800.5192	800.5225	-3.3	PC 38:9	C46H74NO8P
DESI	800.5192	800.5201	-0.9	PC 36:6	C44H76NO8PNa
DESI	800.5192	800.5201	-0.9	PE 39:6	C44H76NO8PNa
DESI	800.5192	800.5202	-1.0	PS O-35:1	C41H80NO9PK
DESI	849.5582	849.5616	-3.4	PG 40:4	C46H83O10PNa
DESI	810.5784	810.5773	1.1	PC O-36:2	C44H86NO7PK
DESI	810.5784	810.5773	1.1	PE O-39:2	C44H86NO7PK
DESI	872.5549	872.5566	-1.7	PC 40:6	C48H84NO8PK
DESI	872.5549	872.5566	-1.7	PE 43:6	C48H84NO8PK
DESI	804.5316	804.5361	-4.5	IPC 34:0;O2	C40H80NO11PNa
DESI	804.5316	804.5304	1.2	PC O-36:5	C44H80NO7PK
DESI	866.6594	866.6633	-3.9	PC 42:4	C50H92NO8P
DESI	866.6594	866.6609	-1.5	PC 40:1	C48H94NO8PNa
DESI	866.6594	866.6609	-1.5	PE 43:1	C48H94NO8PNa
DESI	830.5303	830.5306	-0.3	PE 40:6;O	C45H78NO9PNa
DESI	830.5303	830.5308	-0.5	PS 36:0	C42H82NO10PK
DESI	832.5618	832.5617	0.1	PC O-38:5	C46H84NO7PK
DESI	772.5070	772.5099	-2.9	PS 33:0	C39H76NO10PNa
DESI	810.6081	810.6066	1.5	HexCer 38:1;O4	C44H85NO10Na

DESI	830.5634	830.5670	-3.6	PC 38:5	C46H82NO8PNa
DESI	830.5634	830.5670	-3.6	PE 41:5	C46H82NO8PNa
DESI	830.5634	830.5672	-3.8	PS O-37:0	C43H86NO9PK
DESI	828.5304	828.5304	0.0	PC O-38:7	C46H80NO7PK
DESI	824.5545	824.5552	-0.7	SHexCer 36:1;O3	C42H81NO12S
DESI	824.5545	824.5566	-2.1	PE 39:2	C44H84NO8PK
DESI	824.5545	824.5566	-2.1	PC 36:2	C44H84NO8PK
DESI	874.5547	874.5569	-2.2	PS 41:5	C47H82NO10PNa
DESI	872.5475	872.5496	2.1	Hex2Cer 32:1;O2	C44H83NO13K
DESI	828.5495	828.5538	-4.3	PC 40:9	C48H78NO8P
DESI	828.5495	828.5514	-1.9	PC 38:6	C46H80NO8PNa
DESI	828.5495	828.5514	-1.9	PE 41:6	C46H80NO8PNa
DESI	828.5495	828.5515	-2.0	PS O-37:1	C43H84NO9PK
DESI	830.5483	830.5518	-3.5	IPC 36:1;O2	C42H82NO11PNa
DESI	830.5483	830.5460	2.3	PC O-38:6	C46H82NO7PK
DESI	810.5963	810.6007	-4.4	PC 38:4	C46H84NO8P
DESI	810.5963	810.6007	-4.4	PE 41:4	C46H84NO8P
DESI	810.5963	810.5983	-2.0	PE 39:1	C44H86NO8PNa
DESI	810.5963	810.5983	-2.0	PC 36:1	C44H86NO8PNa
DESI	868.5205	868.5253	-4.8	PC 40:8	C48H80NO8PK
DESI	814.5160	814.5147	1.3	PE O-40:7	C45H78NO7PK
DESI	864.6444	864.6477	-2.3	PC 42:5	C50H90NO8P
DESI	864.6444	864.6407	3.7	Hex2Cer 34:0;O2	C46H89NO13
DESI	864.6444	864.6453	-0.9	PC 40:2	C48H92NO8PNa
DESI	864.6444	864.6453	-0.9	PE 43:2	C48H92NO8PNa
DESI	824.4825	824.4838	-1.3	PS 36:3	C42H76NO10PK
DESI	832.5805	832.5851	-4.6	PC 40:7	C48H82NO8P
DESI	832.5805	832.5827	-2.2	PC 38:4	C46H84NO8PNa

DESI	832.5805	832.5827	-2.2	PE 41:4	C46H84NO8PNa
DESI	804.5497	804.5538	-4.1	PC 38:7	C46H78NO8P
DESI	804.5497	804.5538	-4.1	PE 41:7	C46H78NO8P
DESI	804.5497	804.5514	-1.7	PE 39:4	C44H80NO8PNa
DESI	804.5497	804.5514	-1.7	PC 36:4	C44H80NO8PNa
DESI	806.4901	806.4943	-4.2	PS 36:4	C42H74NO10PNa
DESI	866.6457	866.6481	-2.4	IPC 40:0;O2	C46H92NO11P
DESI	866.6457	866.6482	-2.5	HexCer 42:1;O3	C48H93NO9K
DESI	874.4736	874.4784	-4.8	PE 44:12	C49H74NO8PK
DESI	856.5605	856.5617	-1.2	PC O-40:7	C48H84NO7PK
DESI	782.5000	782.4967	3.3	PS 36:5	C42H72NO10P
DESI	796.5059	796.5099	-4.0	PS 35:2	C41H76NO10PNa
DESI	796.5059	796.5042	1.7	PE dO-40:9	C45H76NO6PK
DESI	810.5267	810.5280	-1.3	PC 36:6;O2	C44H76NO10P
DESI	810.5267	810.5280	. -1.3	PS 38:5	C44H76NO10P
DESI	810.5267	810.5256	1.1	PS 36:2	C42H78NO10PNa
DESI	822.5209	822.5256	-4.7	PS 37:3	C43H78NO10PNa
DESI	850.5326	850.5357	-3.1	PC 40:9	C48H78NO8PNa
DESI	804.4807	804.4810	-0.3	PS 38:8	C44H70NO10P
DESI	804.4807	804.4786	2.1	PS 36:5	C42H72NO10PNa
DESI	808.5627	808.5603	2.4	SHexCer 36:1;O2	C42H81NO11S
DESI	808.5627	808.5617	1.0	PC O-36:3	C44H84NO7PK
DESI	830.4766	830.4733	3.3	PS O-38:7	C44H74NO9PK
DESI	868.5069	868.5101	-3.2	PC 36:4;O3	C44H80NO11PK
DESI	850.5534	850.5569	-3.5	PS 39:3	C45H82NO10PNa
DESI	800.4858	800.4838	2.0	PS 34:1	C40H76NO10PK
DESI	896.5530	896.5566	-3.6	PC 42:8	C50H84NO8PK
DESI	814.5441	814.5440	0.1	IPC 34:0;O4	C40H80NO13P

DESI	830.5068	830.5097	-2.9	PC 37:6	C45H78NO8PK
DESI	830.5068	830.5097	-2.9	PE 40:6	C45H78NO8PK
DESI	830.5068	830.5097	-2.9	PE O-40:7;O	C45H78NO8PK
DESI	864.6298	864.6324	-2.6	IPC 40:1;O2	C46H90NO11P
DESI	864.6298	864.6325	-2.7	HexCer 42:2;O3	C48H91NO9K
DESI	852.6104	852.6113	-0.9	PS O-42:5	C48H86NO9P
DESI	852.6104	852.6089	1.5	PS O-40:2	C46H88NO9PNa
DESI	852.6104	852.6114	-1.0	DGTA 40:5	C50H87NO7K
DESI	806.5493	806.5460	3.3	PC O-36:4	C44H82NO7PK
DESI	866.5063	866.5097	-3.4	PC 40:9	C48H78NO8PK
DESI	808.5494	808.5487	0.7	PE 40:6;O	C45H78NO9P
DESI	808.5494	808.5463	3.1	PS O-37:3	C43H80NO9PNa
DESI	828.4771	828.4810	-3.9	PS 40:10	C46H70NO10P
DESI	828.4771	828.4786	-1.5	PS 38:7	C44H72NO10PNa
DESI	870.5390	870.5410	-2.0	PC 40:7	C48H82NO8PK
DESI	814.5339	814.5381	-4.2	PE 42:9	C47H76NO8P
DESI	814.5339	814.5357	-1.8	PC 37:6	C45H78NO8PNa
DESI	814.5339	814.5357	-1.8	PE 40:6	C45H78NO8PNa
DESI	814.5339	814.5357	-1.8	PE O-40:7;O	C45H78NO8PNa
DESI	814.5339	814.5359	-2.0	PS O-36:1	C42H82NO9PK
DESI	892.5211	892.5253	-4.2	PC 42:10	C50H80NO8PK
DESI	858.5607	858.5621	-1.4	PS 38:0	C44H86NO10PK
DESI	822.5021	822.5044	-2.3	PC 38:9	C46H74NO8PNa
DESI	822.5021	822.5046	-2.5	PE 38:4;O	C43H78NO9PK
DESI	814.4946	814.4993	-4.7	PS O-38:7	C44H74NO9PNa
DESI	814.4946	814.4995	-4.9	PS 35:1	C41H78NO10PK
DESI	838.6265	838.6296	-3.1	PC 38:1	C46H90NO8PNa
DESI	838.6265	838.6296	-3.1	PE 41:1	C46H90NO8PNa

DESI	838.5957	838.5932	2.5	PS O-39:2	C45H86NO9PNa
DESI	852.6422	852.6453	-3.1	PC 39:1	C47H92NO8PNa
DESI	852.6422	852.6453	-3.1	PE 42:1	C47H92NO8PNa
DESI	856.5811	856.5851	-4.0	PC 42:9	C50H82NO8P
DESI	856.5811	856.5827	-1.6	PC 40:6	C48H84NO8PNa
DESI	856.5811	856.5827	-1.6	PE 43:6	C48H84NO8PNa
DESI	856.5811	856.5828	-1.7	PS O-39:1	C45H88NO9PK
DESI	830.4346	830.4369	-2.3	PS 37:7	C43H70NO10PK
DESI	814.5558	814.5593	-3.5	PS 38:3	C44H80NO10P
DESI	814.5558	814.5569	-1.1	PS 36:0	C42H82NO10PNa
DESI	852.6291	852.6243	4.8	PC O-39:2	C47H92NO7PK
DESI	852.6291	852.6243	4.8	PE O-42:2	C47H92NO7PK
DESI	806.5079	806.5097	-1.8	PC 35:4	C43H78NO8PK
DESI	806.5079	806.5097	-1.8	PE 38:4	C43H78NO8PK
DESI	806.5079	806.5097	-1.8	PE O-38:5;O	C43H78NO8PK
DESI	876.5984	876.5936	4.8	IPC 38:0;O3	C44H88NO12PNa
DESI	866.5657	866.5670	-1.3	PE 44:8	C49H82NO8PNa
DESI	866.5657	866.5672	-1.5	PS O-40:3	C46H86NO9PK
DESI	920.5529	920.5566	-3.7	PC 44:10	C52H84NO8PK
DESI	814.4631	814.4630	0.1	PS 37:7	C43H70NO10PNa
DESI	796.5231	796.5239	-0.8	SHexCer 34:1;O3	C40H77NO12S
DESI	796.5231	796.5252	-2.1	PE O-40:8	C45H76NO7PNa
DESI	796.5231	796.5253	-2.2	PC 34:2	C42H80NO8PK
DESI	796.5231	796.5253	-2.2	PE 37:2	C42H80NO8PK
DESI	796.5231	796.5253	-2.2	PE-NMe 36:2	C42H80NO8PK
DESI	802.4589	802.4630	-4.1	PS 36:6	C42H70NO10PNa
DESI	838.6139	838.6168	-2.9	IPC 38:0;O2	C44H88NO11P
DESI	838.6139	838.6169	-3.0	HexCer 40:1;O3	C46H89NO9K

DESI	864.5506	864.5515	-0.9	PC 38:4;O	C46H84NO9PK
DESI	864.5506	864.5515	-0.9	PS O-40:4	C46H84NO9PK
DESI	806.5297	806.5306	-0.9	PE 38:4;O	C43H78NO9PNa
DESI	806.4383	806.4369	1.4	PS 35:5	C41H70NO10PK
DESI	854.5449	854.5460	-1.1	PC O-40:8	C48H82NO7PK
DESI	796.4558	796.4525	3.3	PS 34:3	C40H72NO10PK
DESI	854.5834	854.5882	-4.8	PS 39:1	C45H86NO10PNa
DESI	850.6524	850.6533	-0.9	HexCer 42:1;O2	C48H93NO8K
DESI	818.4742	818.4733	0.9	PE 38:6;O	C43H74NO9PK
DESI	894.5380	894.5410	-3.0	PC 42:9	C50H82NO8PK
DESI	850.4760	850.4784	-2.4	PE 42:10	C47H74NO8PK
DESI	916.5213	916.5253	-4.0	PC 44:12	C52H80NO8PK
DESI	802.5350	802.5381	-3.1	PC 38:8	C46H76NO8P
DESI	802.5350	802.5357	-0.7	PC 36:5	C44H78NO8PNa
DESI	802.5350	802.5357	-0.7	PE 39:5	C44H78NO8PNa
DESI	802.5350	802.5359	-0.9	PS O-35:0	C41H82NO9PK
DESI	880.5439	880.5464	2.5	PS 40:3	C46H84NO10PK
DESI	842.5460	842.5460	0.0	PE O-42:7	C47H82NO7PK
DESI	858.5034	858.5044	-1.0	PE 44:12	C49H74NO8PNa
DESI	858.5034	858.5046	-1.2	PS O-40:7	C46H78NO9PK
DESI	851.6363	851.6403	-4.0	SM 42:2;O2	C47H93N2O6PK
DESI	770.5271	770.5306	-3.5	PS O-34:1	C40H78NO9PNa
DESI	840.4918	840.4940	-2.2	PC 38:8	C46H76NO8PK
DESI	836.6089	836.6094	-0.5	Hex2Cer 32:0;O2	C44H85NO13
DESI	756.5336	756.5304	3.2	PC O-32:1	C40H80NO7PK
DESI	756.5336	756.5304	3.2	PE O-35:1	C40H80NO7PK
DESI	756.5336	756.5386	-5.0	HexCer 34:0;O3	C40H79NO9K
DESI	854.6102	854.6117	-1.5	IPC 38:0;O3	C44H88NO12P

DESI	854.6102	854.6118	-1.6	HexCer 40:1;O4	C46H89NO10K
DESI	852.5522	852.5538	-1.6	PC 42:11	C50H78NO8P
DESI	852.5522	852.5514	0.8	PC 40:8	C48H80NO8PNa
DESI	842.5759	842.5753	0.6	IPC 36:0;O4	C42H84NO13P
DESI	784.5435	784.5463	-2.8	PS O-35:1	C41H80NO9PNa
DESI	878.4925	878.4943	-1.8	PS 42:10	C48H74NO10PNa
DESI	818.5018	818.5059	-4.1	SHexCer 34:1;O3	C40H77NO12SNa
DESI	790.4960	790.4993	-3.3	PS O-36:5	C42H74NO9PNa
DESI	876.5132	876.5151	-1.9	PS 40:5	C46H80NO10PK
DESI	836.5269	836.5225	4.4	PE 44:12	C49H74NO8P
DESI	836.5269	836.5259	1.0	IPC 34:0;O4	C40H80NO13PNa
DESI	838.5396	838.5357	3.9	PC 39:8	C47H78NO8PNa
DESI	838.5396	838.5357	3.9	PE 42:8	C47H78NO8PNa
DESI	838.5396	838.5359	3.7	PS O-38:3	C44H82NO9PK
DESI	812.5649	812.5649	0.0	HexCer 37:1;O4	C43H83NO10K
DESI	812.5014	812.4991	2.3	PE O-40:8	C45H76NO7PK
DESI	703.5622	703.5636	-1.4	PA O-37:1	C40H79O7P
DESI	703.5622	703.5637	-1.5	DG 39:1	C42H80O5K
DESI	858.5404	858.5410	-0.6	PC 39:6	C47H82NO8PK
DESI	858.5404	858.5410	-0.6	PE 42:6	C47H82NO8PK
DESI	790.5179	790.5147	3.2	PE O-38:5	C43H78NO7PK
DESI	788.6148	788.6164	-1.6	PC 36:1	C44H86NO8P
DESI	788.6148	788.6164	-1.6	PE 39:1	C44H86NO8P
DESI	860.5199	860.5202	-0.3	PS O-40:6	C46H80NO9PK
DESI	880.6032	880.6038	-0.6	PS 41:2	C47H88NO10PNa
DESI	862.4984	862.4995	-1.1	PS 39:5	C45H78NO10PK
DESI	912.6135	912.6090	4.5	PS 42:1	C48H92NO10PK
DESI	689.5456	689.5480	-2.4	PA O-36:1	C39H77O7P

DESI	689.5456	689.5481	-2.5	DG 38:1	C41H78O5K
DESI	967.7180	967.7151	2.9	TG 58:9	C61H100O6K
DESI	774.5054	774.5068	-1.4	PC 36:8	C44H72NO8P
DESI	774.5054	774.5068	-1.4	PE 39:8	C44H72NO8P
DESI	774.5054	774.5044	1.0	PC 34:5	C42H74NO8PNa
DESI	774.5054	774.5044	1.0	PE 37:5	C42H74NO8PNa
DESI	774.5054	774.5046	0.8	PS O-33:0	C39H78NO9PK
DESI	880.5796	880.5827	-3.1	PC 42:8	C50H84NO8PNa
DESI	880.6427	880.6402	2.5	PS O-42:2	C48H92NO9PNa
DESI	836.5005	836.5050	-4.5	IPC 34:0;O3	C40H80NO12PK
DESI	770.4918	770.4967	-4.9	PS 35:4	C41H72NO10P
DESI	770.4918	770.4943	-2.5	PS 33:1	C39H74NO10PNa
DESI	770.4918	770.4885	3.3	PE dO-38:8	C43H74NO6PK
DESI	717.5782	717.5793	-1.1	PA O-38:1	C41H81O7P
DESI	717.5782	717.5792	-1.0	ST 46:4;O4	C46H78O4Na
DESI	717.5782	717.5794	-1.2	DG 40:1	C43H82O5K
DESI	880.6240	880.6192	4.8	PC 40:2	C48H92NO8PK
DESI	880.6240	880.6192	4.8	PE 43:2	C48H92NO8PK
DESI	878.6248	878.6245	0.3	PS O-42:3	C48H90NO9PNa
DESI	788.5481	788.5436	4.5	PS 36:2	C42H78NO10P
DESI	786.4650	786.4682	-3.2	PS 33:1	C39H74NO10PK
DESI	731.5948	731.5949	-0.1	PA O-39:1	C42H83O7P
DESI	731.5948	731.5950	-0.2	DG 41:1	C44H84O5K
DESI	900.5111	900.5151	-4.0	PS 42:7	C48H80NO10PK
DESI	747.4977	747.4959	1.8	PA 40:7	C43H71O8P
DESI	747.4977	747.4935	4.2	PA 38:4	C41H73O8PNa
DESI	747.4977	747.4937	4.0	PG O-32:0	C38H77O9PK
DESI	691.4310	691.4333	-2.3	PA 36:7	C39H63O8P

DESI	691.4310	691.4309	0.1	PA 34:4	C37H65O8PNa
DESI	691.4310	691.4311	-0.1	PG O-28:0	C34H69O9PK
DESI	745.6121	745.6106	1.5	PA O-40:1	C43H85O7P
DESI	745.6121	745.6107	1.4	DG 42:1	C45H86O5K
DESI	862.6330	862.6320	1.0	PC 42:6	C50H88NO8P
DESI	862.6330	862.6296	3.4	PC 40:3	C48H90NO8PNa
DESI	753.6038	753.6004	3.4	TG 43:3	C46H82O6Na
DESI	835.6457	835.6423	3.4	PG 40:0	C46H91O10P
DESI	727.5218	727.5248	-3.0	PA 36:0	C39H77O8PNa
DESI	835.6739	835.6786	-4.7	PG O-41:0	C47H95O9P
DESI	835.6739	835.6786	-4.7	TG 49:4	C52H92O6Na
DESI	786.4869	786.4834	3.5	PE O-38:7	C43H74NO7PK
DESI	739.4704	739.4675	2.9	PA 36:2	C39H73O8PK
DESI	719.5590	719.5585	0.5	PA 37:0	C40H79O8P
DESI	719.5590	719.5609	-1.9	DG 44:9	C47H74O5
DESI	719.5590	719.5585	0.5	DG 42:6	C45H76O5Na
DESI	719.5590	719.5586	0.4	TG 39:0	C42H80O6K
MALDI	798.5364	798.5408	-4.4	PE O-40:7	C45H78NO7PNa
MALDI	798.5364	798.5410	-4.6	PC 34:1	C42H82NO8PK
MALDI	798.5364	798.5410	-4.6	PE 37:1	C42H82NO8PK
MALDI	798.5219	798.5256	-3.7	PS 35:1	C41H78NO10PNa
MALDI	798.5219	798.5198	2.1	PE dO-40:8	C45H78NO6PK
MALDI	760.5818	760.5851	-3.3	PC 34:1	C42H82NO8P
MALDI	760.5818	760.5851	-3.3	PE 37:1	C42H82NO8P
MALDI	772.5219	772.5252	-3.3	PE O-38:6	C43H76NO7PNa
MALDI	772.5219	772.5253	-3.4	PC 32:0	C40H80NO8PK
MALDI	772.5219	772.5253	-3.4	PE 35:0	C40H80NO8PK
MALDI	760.5672	760.5639	3.3	PE dO-40:8	C45H78NO6P

MALDI	772.5068	772.5099	-3.1	PS 33:0	C39H76NO10PNa
MALDI	734.5708	734.5694	1.4	PC 32:0	C40H80NO8P
MALDI	734.5708	734.5694	1.4	PE 35:0	C40H80NO8P
MALDI	826.5687	826.5721	-3.4	PE O-42:7	C47H82NO7PNa
MALDI	826.5687	826.5723	-3.6	PE 39:1	C44H86NO8PK
MALDI	826.5687	826.5723	-3.6	PC 36:1	C44H86NO8PK
MALDI	782.5673	782.5694	-2.1	PE 39:4	C44H80NO8P
MALDI	782.5673	782.5694	-2.1	PC 36:4	C44H80NO8P
MALDI	782.5673	782.5670	0.3	PC 34:1	C42H82NO8PNa
MALDI	782.5673	782.5670	0.3	PE 37:1	C42H82NO8PNa
MALDI	844.5248	844.5253	-0.5	PC 38:6	C46H80NO8PK
MALDI	844.5248	844.5253	-0.5	PE 41:6	C46H80NO8PK
MALDI	820.5224	820.5253	-2.9	PC 36:4	C44H80NO8PK
MALDI	820.5224	820.5253	-2.9	PE 39:4	C44H80NO8PK
MALDI	806.5688	806.5694	-0.6	PC 38:6	C46H80NO8P
MALDI	806.5688	806.5694	-0.6	PE 41:6	C46H80NO8P
MALDI	806.5688	806.5670	1.8	PC 36:3	C44H82NO8PNa
MALDI	806.5688	806.5670	1.8	PE 39:3	C44H82NO8PNa
MALDI	810.5967	810.6007	-4.0	PC 38:4	C46H84NO8P
MALDI	810.5967	810.6007	-4.0	PE 41:4	C46H84NO8P
MALDI	810.5967	810.5983	-1.6	PC 36:1	C44H86NO8PNa
MALDI	810.5967	810.5983	-1.6	PE 39:1	C44H86NO8PNa
MALDI	848.5532	848.5566	-3.4	PC 38:4	C46H84NO8PK
MALDI	848.5532	848.5566	-3.4	PE 41:4	C46H84NO8PK
MALDI	788.6152	788.6164	-1.2	PC 36:1	C44H86NO8P
MALDI	788.6152	788.6164	-1.2	PE 39:1	C44H86NO8P
MALDI	782.5487	782.5459	2.8	PE dO-40:8	C45H78NO6PNa
MALDI	782.5487	782.5460	2.7	PC O-34:2	C42H82NO7PK

MALDI	782.5487	782.5460	2.7	PE O-37:2	C42H82NO7PK
MALDI	734.5528	734.5541	-1.3	HexCer 35:2;O2	C41H77NO8Na
MALDI	800.5377	800.5412	-3.5	PS 35:0	C41H80NO10PNa
MALDI	760.5246	760.5252	-0.6	PC O-34:5	C42H76NO7PNa
MALDI	769.5594	769.5620	-2.6	EPC 39:1;O2	C41H83N2O6PK
MALDI	769.5594	769.5620	-2.6	SM 36:1;O2	C41H83N2O6PK
MALDI	830.5083	830.5097	-1.4	PC 37:6	C45H78NO8PK
MALDI	830.5083	830.5097	-1.4	PE 40:6	C45H78NO8PK
MALDI	830.5083	830.5097	-1.4	PE O-40:7;O	C45H78NO8PK
MALDI	872.5563	872.5566	-0.3	PC 40:6	C48H84NO8PK
MALDI	872.5563	872.5566	-0.3	PE 43:6	C48H84NO8PK
MALDI	806.5494	806.5460	3.4	PC O-36:4	C44H82NO7PK
MALDI	810.5794	810.5773	2.1	PC O-36:2	C44H86NO7PK
MALDI	810.5794	810.5773	2.1	PE O-39:2	C44H86NO7PK
MALDI	834.5995	834.6007	-1.2	PC 40:6	C48H84NO8P
MALDI	834.5995	834.6007	-1.2	PE 43:6	C48H84NO8P
MALDI	834.5995	834.5983	1.2	PC 38:3	C46H86NO8PNa
MALDI	834.5995	834.5983	1.2	PE 41:3	C46H86NO8PNa
MALDI	798.4271	798.4317	-4.6	PS 36:8	C42H66NO10PNa
MALDI	826.5011	826.4995	1.6	PS 36:2	C42H78NO10PK
MALDI	824.5542	824.5552	-1.0	SHexCer 36:1;O3	C42H81NO12S
MALDI	824.5542	824.5566	-2.4	PC 36:2	C44H84NO8PK
MALDI	824.5542	824.5566	-2.4	PE 39:2	C44H84NO8PK
MALDI	756.5502	756.5538	-3.6	PC 34:3	C42H78NO8P
MALDI	756.5502	756.5538	-3.6	PE 37:3	C42H78NO8P
MALDI	756.5502	756.5514	-1.2	PC 32:0	C40H80NO8PNa
MALDI	756.5502	756.5514	-1.2	PE 35:0	C40H80NO8PNa
MALDI	739.4662	739.4675	-1.3	PA 36:2	C39H73O8PK

MALDI	844.4511	844.4525	-1.4	PS 38:7	C44H72NO10PK
MALDI	734.5129	734.5177	-4.8	HexCer 34:3;O3	C40H73NO9Na
MALDI	820.4550	820.4525	2.5	PS 36:5	C42H72NO10PK
MALDI	731.6061	731.6061	0.0	EPC 39:1;O2	C41H83N2O6P
MALDI	731.6061	731.6061	0.0	SM 36:1;O2	C41H83N2O6P
MALDI	814.5138	814.5147	-0.9	PE O-40:7	C45H78NO7PK
MALDI	834.5793	834.5773	2.0	PC O-38:4	C46H86NO7PK
MALDI	832.5819	832.5851	-3.2	PC 40:7	C48H82NO8P
MALDI	832.5819	832.5827	-0.8	PC 38:4	C46H84NO8PNa
MALDI	832.5819	832.5827	-0.8	PE 41:4	C46H84NO8PNa
MALDI	824.5454	824.5436	1.8	PS 39:5	C45H78NO10P
MALDI	824.5454	824.5412	4.2	PS 37:2	C43H80NO10PNa
MALDI	786.5985	786.6007	-2.2	PC 36:2	C44H84NO8P
MALDI	786.5985	786.6007	-2.2	PE 39:2	C44H84NO8P
MALDI	848.4816	848.4838	-2.2	PC 36:6;O2	C44H76NO10PK
MALDI	848.4816	848.4838	-2.2	PS 38:5	C44H76NO10PK
MALDI	806.4895	806.4943	-4.8	PS 36:4	C42H74NO10PNa
MALDI	810.5315	810.5280	3.5	PC 36:6;O2	C44H76NO10P
MALDI	810.5315	810.5280	3.5	PS 38:5	C44H76NO10P
MALDI	769.5001	769.5014	-1.3	PG 36:5	C42H73O10P
MALDI	769.5001	769.4990	1.1	LBPA 34:2	C40H75O10PNa
MALDI	769.5001	769.4990	1.1	PG 34:2	C40H75O10PNa
MALDI	874.4986	874.4995	-0.9	PS 40:6	C46H78NO10PK
MALDI	796.5239	796.5239	0.0	SHexCer 34:1;O3	C40H77NO12S
MALDI	796.5239	796.5252	-1.3	PE O-40:8	C45H76NO7PNa
MALDI	796.5239	796.5253	-1.4	PC 34:2	C42H80NO8PK
MALDI	796.5239	796.5253	-1.4	PE-NMe 36:2	C42H80NO8PK
MALDI	796.5239	796.5253	-1.4	PE 37:2	C42H80NO8PK

MALDI	804.5504	804.5538	-3.4	PC 38:7	C46H78NO8P
MALDI	804.5504	804.5538	-3.4	PE 41:7	C46H78NO8P
MALDI	804.5504	804.5514	-1.0	PE 39:4	C44H80NO8PNa
MALDI	804.5504	804.5514	-1.0	PC 36:4	C44H80NO8PNa
MALDI	713.4520	713.4518	0.2	PA 34:1	C37H71O8PK
MALDI	786.5814	786.5773	4.1	PC O-34:0	C42H86NO7PK
MALDI	786.5814	786.5773	4.1	PE O-37:0	C42H86NO7PK
MALDI	852.4906	852.4940	-3.4	PE 42:9	C47H76NO8PK
MALDI	784.5230	784.5253	-2.3	PC 33:1	C41H80NO8PK
MALDI	784.5230	784.5253	-2.3	PE 36:1	C41H80NO8PK
MALDI	784.5230	784.5253	-2.3	PE-NMe2 34:1	C41H80NO8PK
MALDI	801.5231	801.5195	3.6	PA O-42:6	C45H79O7PK
MALDI	802.4782	802.4784	-0.2	PC 35:6	C43H74NO8PK
MALDI	802.4782	802.4784	-0.2	PE 38:6	C43H74NO8PK
MALDI	802.4782	802.4784	-0.2	PE O-38:7;O	C43H74NO8PK
MALDI	866.6479	866.6481	-0.2	IPC 40:0;O2	C46H92NO11P
MALDI	866.6479	866.6482	-0.3	HexCer 42:1;O3	C48H93NO9K
MALDI	790.5132	790.5147	-1.5	PE O-38:5	C43H78NO7PK
MALDI	832.5619	832.5617	0.2	PC O-38:5	C46H84NO7PK
MALDI	864.6316	864.6324	-0.8	IPC 40:1;O2	C46H90NO11P
MALDI	864.6316	864.6325	-0.9	HexCer 42:2;O3	C48H91NO9K
MALDI	796.5057	796.5099	-4.2	PS 35:2	C41H76NO10PNa
MALDI	796.5057	796.5042	1.5	PE dO-40:9	C45H76NO6PK
MALDI	834.5282	834.5280	0.2	PS 40:7	C46H76NO10P
MALDI	834.5282	834.5256	-2.6	PC 36:5;O2	C44H78NO10PNa
MALDI	834.5282	834.5256	-2.6	PS 38:4	C44H78NO10PNa
MALDI	822.5206	822.5256	-5.0	PS 37:3	C43H78NO10PNa
MALDI	804.5314	804.5361	-4.7	IPC 34:0;O2	C40H80NO11PNa

MALDI	804.5314	804.5304	1.0	PC O-36:5	C44H80NO7PK
MALDI	828.5304	828.5304	0.0	PC O-38:7	C46H80NO7PK
MALDI	828.4906	828.4940	-3.4	PC 37:7	C45H76NO8PK
MALDI	828.4906	828.4940	-3.4	PE 40:7	C45H76NO8PK
MALDI	963.4780	963.4761	1.9	CL 36:4	C45H82O15P2K
MALDI	870.5411	870.5410	0.1	PC 40:7	C48H82NO8PK
MALDI	826.5185	826.5147	3.8	PC O-38:8	C46H78NO7PK
MALDI	830.4376	830.4369	0.7	PS 37:7	C43H70NO10PK
MALDI	762.5399	762.5408	0.9	PC O-34:4	C42H78NO7PNa
MALDI	762.5399	762.5408	0.9	PE O-37:4	C42H78NO7PNa
MALDI	925.5209	925.5202	0.7	CL 36:4	C45H82O15P2
MALDI	925.5209	925.5203	0.6	Glc-GP 38:4	C47H83O13PK
MALDI	925.5209	925.5203	0.6	PI 38:4	C47H83O13PK
MALDI	874.4758	874.4784	-2.6	PE 44:12	C49H74NO8PK
MALDI	753.5866	753.5881	-1.5	EPC 39:1;O2	C41H83N2O6PNa
MALDI	753.5866	753.5881	-1.5	SM 36:1;O2	C41H83N2O6PNa
MALDI	770.4914	770.4943	-2.9	PS 33:1	C39H74NO10PNa
MALDI	770.4914	770.4885	-2.9	PE dO-38:8	C43H74NO6PK
MALDI	782.5212	782.5179	3.3	HexCer 35:2;O4	C41H77NO10K
MALDI	824.4856	824.4838	1.8	PS 36:3	C42H76NO10PK
MALDI	767.4973	767.4988	1.5	PA 38:2	C41H77O8PK
MALDI	785.4510	785.4518	-0.8	PA 40:7	C43H71O8PK
MALDI	896.4813	896.4838	-2.5	PS 42:9	C48H76NO10PK
MALDI	734.4731	734.4755	-2.4	PE 36:7	C41H68NO8P
MALDI	734.4731	734.4731	0.0	PC 31:4	C39H70NO8PNa
MALDI	734.4731	734.4731	0.0	PE 34:4	C39H70NO8PNa
MALDI	851.6391	851.6403	-1.2	SM 42:2;O2	C47H93N2O6PK
MALDI	756.4913	756.4940	-2.7	PC 31:1	C39H76NO8PK

MALDI	756.4913	756.4940	-2.7	PE 34:1	C39H76NO8PK
MALDI	834.5384	834.5410	-2.6	PC 37:4	C45H82NO8PK
MALDI	834.5384	834.5410	-2.6	PE 40:4	C45H82NO8PK
MALDI	814.5337	814.5381	-4.4	PE 42:9	C47H76NO8P
MALDI	814.5337	814.5357	-2.0	PC 37:6	C45H78NO8PNa
MALDI	814.5337	814.5357	-2.0	PE 40:6	C45H78NO8PNa
MALDI	814.5337	814.5357	-2.0	PE O-40:7;O	C45H78NO8PNa
MALDI	814.5337	814.5359	-2.2	PS O-36:1	C42H82NO9PK
MALDI	802.4590	802.4630	-4.0	PS 36:6	C42H70NO10PNa
MALDI	838.6126	838.6168	-4.2	IPC 38:0;O2	C44H88NO11P
MALDI	838.6126	838.6086	4.0	PC O-38:2	C46H90NO7PK
MALDI	838.6126	838.6169	-4.3	HexCer 40:1;O3	C46H89NO9K
MALDI	758.5673	758.5694	-2.1	PE-NMe 36:2	C42H80NO8P
MALDI	758.5673	758.5694	-2.1	PE 37:2	C42H80NO8P
MALDI	758.5673	758.5694	-2.1	PC 34:2	C42H80NO8P
MALDI	774.5988	774.6007	-1.9	PC 35:1	C43H84NO8P
MALDI	774.5988	774.6007	-1.9	PE 38:1	C43H84NO8P
MALDI	812.5552	812.5565	-1.3	PC O-38:7	C46H80NO7PNa
MALDI	812.5552	812.5566	-1.4	PC 35:1	C43H84NO8PK
MALDI	812.5552	812.5566	-1.4	PE 38:1	C43H84NO8PK
MALDI	856.5827	856.5851	-2.4	PC 42:9	C50H82NO8P
MALDI	856.5827	856.5827	0.0	PC 40:6	C48H84NO8PNa
MALDI	856.5827	856.5827	0.0	PE 43:6	C48H84NO8PNa
MALDI	856.5827	856.5828	-0.1	PS O-39:1	C45H88NO9PK
MALDI	806.4417	806.4369	4.8	PS 35:5	C41H70NO10PK
MALDI	732.5530	732.5538	-0.8	PC 32:1	C40H78NO8P
MALDI	732.5530	732.5538	-0.8	PE 35:1	C40H78NO8P
MALDI	732.5530	732.5538	-0.8	PE-NMe 34:1	C40H78NO8P

MALDI	789.4809	789.4831	-2.2	PA 40:5	C43H75O8PK
MALDI	761.4503	761.4518	-1.5	PA 38:5	C41H71O8PK
MALDI	810.5484	810.5492	-0.8	HexCer 37:2;O4	C43H81NO10K
MALDI	864.6207	864.6229	-2.2	SHexCer 40:1;O2	C46H89NO11S
MALDI	864.6207	864.6243	-3.6	PC O-40:3	C48H92NO7PK
MALDI	786.4816	786.4834	-1.8	PE O-38:7	C43H74NO7PK
MALDI	767.4809	767.4858	-4.9	PG 36:6	C42H71O10P
MALDI	767.4809	767.4834	-2.5	PG 34:3	C40H73O10PNa
MALDI	810.4834	810.4834	0.0	PE O-40:9	C45H74NO7PK
MALDI	818.5424	818.5460	-3.6	PE O-40:5	C45H82NO7PK
MALDI	804.4842	804.4810	3.2	PS 38:8	C44H70NO10P
MALDI	767.5441	767.5464	-2.3	EPC 39:2;O2	C41H81N2O6PK
MALDI	767.5441	767.5464	-2.3	SM 36:2;O2	C41H81N2O6PK
MALDI	767.5279	767.5304	-2.5	GlcADG 34:3	C43H74O11
MALDI	767.5279	767.5304	-2.5	MGDG 34:4;O	C43H74O11
MALDI	775.5084	775.5038	4.6	PA O-40:5	C43H77O7PK
MALDI	774.5823	774.5796	2.7	PC dO-38:8	C46H80NO6P
MALDI	774.5823	774.5854	-3.1	HexCer 38:3;O2	C44H81NO8Na
MALDI	789.4637	789.4677	-4.0	PG 36:6	C42H71O10PNa
MALDI	813.4828	813.4831	-0.3	PA 42:7	C45H75O8PK
MALDI	876.5864	876.5879	-1.5	PC 40:4	C48H88NO8PK
MALDI	876.5864	876.5879	-1.5	PE 43:4	C48H88NO8PK
MALDI	845.6618	845.6630	-1.2	PG O-42:2	C48H93O9P
MALDI	845.6618	845.6654	-3.6	TG 52:9	C55H88O6
MALDI	845.6618	845.6606	1.2	PG dO-40:10;1	C46H95O9PNa
MALDI	845.6618	845.6630	-1.2	TG 50:6	C53H90O6Na
MALDI	838.6295	838.6320	-2.5	PC 40:4	C48H88NO8P
MALDI	838.6295	838.6320	-2.5	PE 43:4	C48H88NO8P

MALDI	838.6295	838.6296	-0.1	PC 38:1	C46H90NO8PNa
MALDI	838.6295	838.6296	-0.1	PE 41:1	C46H90NO8PNa
MALDI	785.4331	785.4364	-3.3	PG 36:8	C42H67O10PNa
MALDI	758.5085	758.5097	-1.2	PC 31:0	C39H78NO8PK
MALDI	758.5085	758.5097	-1.2	PE 34:0	C39H78NO8PK
MALDI	758.5085	758.5097	-1.2	PE-NMe2 32:0	C39H78NO8PK
MALDI	874.5502	874.5475	2.7	SHexCer 38:1;O2	C44H85NO11SK
MALDI	723.4928	723.4959	-3.1	PA 38:5	C41H71O8P
MALDI	723.4928	723.4935	-0.7	PA 36:2	C39H73O8PNa
MALDI	824.4614	824.4627	-1.3	PE 40:9	C45H72NO8PK
MALDI	741.4812	741.4831	-1.9	PA 36:1	C39H75O8PK
MALDI	947.5038	947.5021	1.7	CL 36:4	C45H82O15P2Na
MALDI	947.5038	947.5046	-0.8	PI 40:7	C49H81O13PK
MALDI	813.6820	813.6844	-2.4	SM 42:2;O2	C47H93N2O6P
MALDI	852.6105	852.6113	-0.8	PS O-42:5	C48H86NO9P
MALDI	852.6105	852.6089	1.6	PS O-40:2	C46H88NO9PNa
MALDI	852.6105	852.6114	-0.9	DGTA 40:5	C50H87NO7K
MALDI	766.5139	766.5147	-0.8	PC O-33:3	C41H78NO7PK
MALDI	766.5139	766.5147	-0.8	PE O-36:3	C41H78NO7PK
MALDI	848.6130	848.6164	-3.4	PC 41:6	C49H86NO8P
MALDI	848.6130	848.6164	-3.4	PE 44:6	C49H86NO8P
MALDI	848.6130	848.6140	-1.0	PC 39:3	C47H88NO8PNa
MALDI	848.6130	848.6140	-1.0	PE 42:3	C47H88NO8PNa
MALDI	697.4789	697.4803	-1.4	PA 36:4	C39H69O8P
MALDI	697.4789	697.4779	1.0	PA 34:1	C37H71O8PNa
MALDI	748.5849	748.5851	-0.2	PC 33:0	C41H82NO8P
MALDI	748.5849	748.5851	-0.2	PE 36:0	C41H82NO8P
MALDI	732.5375	732.5326	4.9	PE dO-38:8	C43H74NO6P

MALDI	732.5375	732.5385	-1.0	HexCer 35:3;O2	C41H75NO8Na
MALDI	792.5523	792.5538	-1.5	PC 37:6	C45H78NO8P
MALDI	792.5523	792.5538	-1.5	PE 40:6	C45H78NO8P
MALDI	792.5523	792.5538	-1.5	PE O-40:7;O	C45H78NO8P
MALDI	792.5523	792.5514	0.9	PC 35:3	C43H80NO8PNa
MALDI	792.5523	792.5514	0.9	PE 38:3	C43H80NO8PNa
MALDI	746.6056	746.6058	-0.2	PC O-34:1	C42H84NO7P
MALDI	746.6056	746.6058	-0.2	PE O-37:1	C42H84NO7P
MALDI	858.5241	858.5280	-3.9	PS 42:9	C48H76NO10P
MALDI	858.5241	858.5256	-1.5	PS 40:6	C46H78NO10PNa
MALDI	813.4633	813.4677	-4.4	PG 38:8	C44H71O10PNa
MALDI	790.5346	790.5381	-3.5	PC 37:7	C45H76NO8P
MALDI	790.5346	790.5381	-3.5	PE 40:7	C45H76NO8P
MALDI	790.5346	790.5357	-1.1	PC 35:4	C43H78NO8PNa
MALDI	790.5346	790.5357	-1.1	PE 38:4	C43H78NO8PNa
MALDI	790.5346	790.5357	-1.1	PE O-38:5;O	C43H78NO8PNa
MALDI	758.4926	758.4967	-4.1	PS 34:3	C40H72NO10P
MALDI	758.4926	758.4943	-1.7	PS 32:0	C38H74NO10PNa
MALDI	866.5702	866.5670	3.2	PE 44:8	C49H82NO8PNa
MALDI	866.5702	866.5672	3.0	PS O-40:3	C46H86NO9PK
MALDI	864.5548	864.5572	-2.4	IPC 36:0;O4	C42H84NO13PNa
MALDI	864.5548	864.5515	3.3	PC 38:4;O	C46H84NO9PK
MALDI	864.5548	864.5515	3.3	PS O-40:4	C46H84NO9PK
MALDI	852.5869	852.5865	0.4	SHexCer 38:1;O3	C44H85NO12S
MALDI	852.5869	852.5879	-1.0	PC 38:2	C46H88NO8PK
MALDI	852.5869	852.5879	-1.0	PE 41:2	C46H88NO8PK
MALDI	830.5552	830.5542	1.0	PC 36:4;O3	C44H80NO11P
MALDI	830.5552	830.5518	3.4	IPC 36:1;O2	C42H82NO11PNa

MALDI	729.5911	729.5905	0.6	EPC 39:2;O2	C41H81N2O6P
MALDI	729.5911	729.5905	0.6	SM 36:2;O2	C41H81N2O6P
MALDI	854.6025	854.6036	-1.1	PC 38:1	C46H90NO8PK
MALDI	854.6025	854.6036	-1.1	PE 41:1	C46H90NO8PK
MALDI	780.5504	780.5538	-3.4	PC 36:5	C44H78NO8P
MALDI	780.5504	780.5538	-3.4	PE 39:5	C44H78NO8P
MALDI	780.5504	780.5514	-1.0	PC 34:2	C42H80NO8PNa
MALDI	780.5504	780.5514	-1.0	PE 37:2	C42H80NO8PNa
MALDI	780.5504	780.5514	-1.0	PE-NMe 36:2	C42H80NO8PNa
MALDI	748.5685	748.5698	-1.3	HexCer 36:2;O2	C42H79NO8Na
MALDI	786.5052	786.5068	-1.6	PE 40:9	C45H72NO8P
MALDI	786.5052	786.5044	0.8	PC 35:6	C43H74NO8PNa
MALDI	786.5052	786.5044	0.8	PE 38:6	C43H74NO8PNa
MALDI	786.5052	786.5044	0.8	PE O-38:7;O	C43H74NO8PNa
MALDI	786.5052	786.5046	0.6	PS O-34:1	C40H78NO9PK
MALDI	753.5283	753.5307	-2.4	EPC 38:2;O2	C40H79N2O6PK
MALDI	766.4973	766.5017	4.4	PS O-36:6	C42H72NO9P
MALDI	766.4973	766.4993	-2.0	PS O-34:3	C40H74NO9PNa
MALDI	909.5474	909.5488	-1.4	PI 40:7	C49H81O13P
MALDI	909.5474	909.5463	1.1	Glc-GP 38:4	C47H83O13PNa
MALDI	909.5474	909.5463	1.1	PI 38:4	C47H83O13PNa
MALDI	778.4754	778.4784	-3.0	PC 33:4	C41H74NO8PK
MALDI	778.4754	778.4784	-3.0	PE 36:4	C41H74NO8PK
MALDI	778.4754	778.4784	-3.0	PE O-36:5;O	C41H74NO8PK
MALDI	854.5683	854.5694	-1.1	PC 42:10	C50H80NO8P
MALDI	854.5683	854.5670	1.3	PC 40:7	C48H82NO8PNa
MALDI	854.5683	854.5672	1.1	PS O-39:2	C45H86NO9PK
MALDI	836.5176	836.5225	-4.9	PE 44:12	C49H74NO8P

MALDI	836.5176	836.5201	-2.5	PE 42:9	C47H76NO8PNa
MALDI	836.5176	836.5202	-2.6	PC 36:4;O	C44H80NO9PK
MALDI	836.5176	836.5202	-2.6	PS O-38:4	C44H80NO9PK
MALDI	792.5341	792.5304	3.7	PC O-35:4	C43H80NO7PK
MALDI	792.5341	792.5304	3.7	PE O-38:4	C43H80NO7PK
MALDI	792.5341	792.5386	-4.5	HexCer 37:3;O3	C43H79NO9K
MALDI	776.5602	776.5589	1.3	PE O-40:7	C45H78NO7P
MALDI	776.5602	776.5565	3.7	PC O-35:4	C43H80NO7PNa
MALDI	776.5602	776.5565	3.7	PE O-38:4	C43H80NO7PNa
MALDI	776.5602	776.5647	-4.5	HexCer 37:3;O3	C43H79NO9Na
MALDI	746.5694	746.5694	0.0	PC 33:1	C41H80NO8P
MALDI	746.5694	746.5694	0.0	PE 36:1	C41H80NO8P
MALDI	746.5694	746.5694	0.0	PE-NMe2 34:1	C41H80NO8P
MALDI	751.3111	751.3067	4.4	PI 23:2;O2	C32H57O15PK
MALDI	856.5233	856.5253	2.0	PC 39:7	C47H80NO8PK
MALDI	856.5233	856.5253	2.0	PE 42:7	C47H80NO8PK
MALDI	858.5413	858.5410	0.3	PC 39:6	C47H82NO8PK
MALDI	858.5413	858.5410	0.3	PE 42:6	C47H82NO8PK
MALDI	916.6558	916.6556	0.2	PC O-44:5	C52H96NO7PK
MALDI	688.3947	688.3950	-0.3	LPS O-27:2;O	C33H64NO9PK
MALDI	688.3947	688.3950	-0.3	PC 25:1;O	C33H64NO9PK
MALDI	765.4651	765.4701	-5.0	PG 36:7	C42H69O10P
MALDI	765.4651	765.4677	-2.6	PG 34:4	C40H71O10PNa
MALDI	853.6540	853.6559	-1.9	SM 42:1;O2	C47H95N2O6PK
MALDI	744.4932	744.4963	-3.1	PE O-38:9	C43H70NO7P
MALDI	744.4932	744.4939	-0.7	PE O-36:6	C41H72NO7PNa
MALDI	744.4932	744.4940	-0.8	PC 30:0	C38H76NO8PK
MALDI	744.4932	744.4940	-0.8	PE 33:0	C38H76NO8PK

MALDI	744.4932	744.4940	-0.8	PE-NMe 32:0	C38H76NO8PK
MALDI	802.6301	802.6320	1.9	PC 37:1	C45H88NO8P
MALDI	802.6301	802.6320	1.9	PE 40:1	C45H88NO8P
MALDI	850.6737	850.6742	-0.5	HexCer 42:1;O3	C48H93NO9Na
MALDI	860.6143	860.6164	-2.1	PC 42:7	C50H86NO8P
MALDI	860.6143	860.6140	0.3	PC 40:4	C48H88NO8PNa
MALDI	860.6143	860.6140	0.3	PE 43:4	C48H88NO8PNa
MALDI	741.5303	741.5307	-0.4	EPC 37:1;O2	C39H79N2O6PK
MALDI	741.5303	741.5307	-0.4	SM 34:1;O2	C39H79N2O6PK
MALDI	763.5579	763.5612	-3.3	PA O-40:3	C43H81O7PNa
MALDI	751.3305	751.3276	2.9	PI 23:2;O3	C32H57O16PNa
MALDI	918.4652	918.4682	-3.0	PS 44:12	C50H74NO10PK
MALDI	706.5398	706.5381	1.7	PC 30:0	C38H76NO8P
MALDI	706.5398	706.5381	1.7	PE 33:0	C38H76NO8P
MALDI	706.5398	706.5381	1.7	PE-NMe 32:0	C38H76NO8P
MALDI	700.4273	700.4314	-4.1	PC 27:1	C35H68NO8PK
MALDI	700.4273	700.4314	-4.1	PE 30:1	C35H68NO8PK
MALDI	880.5087	880.5123	-3.6	PS 44:12	C50H74NO10P
MALDI	880.5087	880.5099	-1.2	PS 42:9	C48H76NO10PNa
MALDI	878.6487	878.6482	0.5	HexCer 43:2;O3	C49H93NO9K
MALDI	928.5962	928.5944	1.8	SHexCer 42:2;O2	C48H91NO11SK
MALDI	751.5236	751.5272	-3.6	PA 40:5	C43H75O8P
MALDI	751.5236	751.5248	-1.2	PA 38:2	C41H77O8PNa
MALDI	699.4194	699.4208	-1.4	PG 29:2	C35H65O10PNa
MALDI	703.5765	703.5748	1.7	EPC 37:1;O2	C39H79N2O6P
MALDI	703.5765	703.5748	1.7	SM 34:1;O2	C39H79N2O6P
MALDI	744.4776	744.4810	-3.4	PS 33:3	C39H70NO10P
MALDI	744.4776	744.4786	-1.0	PS 31:0	C37H72NO10PNa

MALDI	716.4236	716.4263	-2.7	LPS O-29:2;O	C35H68NO9PK
MALDI	716.4236	716.4263	-2.7	PS O-29:1	C35H68NO9PK
MALDI	704.3910	704.3899	1.1	PC 25:1;O2	C33H64NO10PK
MALDI	704.3910	704.3899	1.1	PS 27:0	C33H64NO10PK
MALDI	780.4904	780.4940	-3.6	PC 33:3	C41H76NO8PK
MALDI	780.4904	780.4940	-3.6	PE 36:3	C41H76NO8PK
MALDI	678.4709	678.4704	0.5	LPS O-29:2;O	C35H68NO9P
MALDI	678.4709	678.4704	0.5	PS O-29:1	C35H68NO9P
MALDI	749.2958	749.2910	4.8	PI 23:3;O2	C32H55O15PK
MALDI	878.5307	878.5308	-0.1	PS 40:4	C46H82NO10PK
MALDI	890.6395	890.6386	0.9	SHexCer 42:2;O2	C48H91NO11S
MALDI	890.6395	890.6399	0.9	PC O-42:4	C50H94NO7PK
MALDI	982.5487	982.5529	-4.2	CDP-DG 34:0	C46H85N3O15P2
MALDI	720.5556	720.5538	1.8	PC 31:0	C39H78NO8P
MALDI	720.5556	720.5538	1.8	PE 34:0	C39H78NO8P
MALDI	720.5556	720.5538	1.8	PE-NMe2 32:0	C39H78NO8P
MALDI	711.4389	711.4362	2.7	PA 34:2	C37H69O8PK
MALDI	749.3159	749.3120	3.9	PI 23:3;O3	C32H55O16PNa
MALDI	815.7004	815.7000	0.4	SM 42:1;O2	C47H95N2O6P

Supplementary Table S3: Sutter Instruments P-2000 micropipette puller program for the tip pulled from 100µm inner diameter by 200µm outer diameter fused silica. For the tip shown the program was looped one time, the last line used was line three with heat on for 2.27 seconds.

HEAT	FIL	VEL	DELAY	PULL
280	0	25	180	25
250	0	25	180	25
250	0	25	180	25
